# SELEX based aptamers with diagnostic and entry inhibitor therapeutic potential for SARS-CoV-2

**DOI:** 10.1038/s41598-023-41885-w

**Published:** 2023-09-04

**Authors:** Sayanti Halder, Abhishek Thakur, Supriya Suman Keshry, Pradip Jana, Divyanshi Karothia, Indrani Das Jana, Orlando Acevedo, Rajeeb K. Swain, Arindam Mondal, Soma Chattopadhyay, Venkatesan Jayaprakash, Abhimanyu Dev

**Affiliations:** 1 Department of Pharmaceutical Sciences and Technology, Birla Institute of Technology, Mesra, Ranchi, Jharkhand 835215 India; 2 Department of Chemistry, University of Miami, Coral Gables, FL 33146 USA; 3 Institute of Life Sciences, Bhubaneswar, Odisha 751023 India; 4 School of Bioscience, Indian Institute of Technology Kharagpur, Kharagpur, West Bengal 721302 India; 5 School of Biotechnology, Kalinga Institute of Industrial Technology (KIIT) University, Bhubaneswar, Odisha India

**Keywords:** Drug discovery, Molecular biology, Molecular medicine

## Abstract

Frequent mutation and variable immunological protection against vaccination is a common feature for COVID-19 pandemic. Early detection and confinement remain key to controlling further spread of infection. In response, we have developed an aptamer-based system that possesses both diagnostic and therapeutic potential towards the virus. A random aptamer library (~ 10^17^ molecules) was screened using systematic evolution of ligands by exponential enrichment (SELEX) and aptamer R was identified as a potent binder for the SARS-CoV-2 spike receptor binding domain (RBD) using in vitro binding assay. Using a pseudotyped viral entry assay we have shown that aptamer R specifically inhibited the entry of a SARS-CoV-2 pseudotyped virus in HEK293T-ACE2 cells but did not inhibit the entry of a Vesicular Stomatitis Virus (VSV) glycoprotein (G) pseudotyped virus, hence establishing its specificity towards SARS-CoV-2 spike protein. The antiviral potential of aptamers R and J (same central sequence as R but lacking flanked primer regions) was tested and showed 95.4% and 82.5% inhibition, respectively, against the SARS-CoV-2 virus. Finally, intermolecular interactions between the aptamers and the RBD domain were analyzed using in silico docking and molecular dynamics simulations that provided additional insight into the binding and inhibitory action of aptamers R and J.

## Introduction

The sudden outbreak of COVID-19 infection in 2019 not only challenged the global healthcare system but also drastically affected the economic conditions across the world. According to WHO statistics to date, the SARS-CoV-2 virus has affected ~ 600 M people with a mortality rate of more than 1% (6.48 M)^1^. The emergence of variant strains (omicron, delta virus, etc.) has further aggravated the situation^2–4^. Fortunately, the ongoing epidemic has been partially controlled with the launch of vaccines that provide substantial protection against the SARS-CoV-2 virus and mutants. However, major challenges still exist, e.g., the logistics of vaccinating the world population including the least developed nations and the length of protection duration. For example, a published report by Feikin et al. in *The Lancet* found the effectiveness of protection by various vaccines (Pfizer–BioNTech-Comirnaty, n = 38; Moderna-mRNA-1273, n = 23; Janssen-Ad26.COV2.S, n = 9; and AstraZeneca-Vaxzevria, n = 8) decreased by 21 percentage points in a duration from 1 to 6 months after full vaccination^5^. Early detection and confinement remain key to controlling the massive spread of an infection. As such, robust and specific diagnostic kits that nullify false diagnosis are of the upmost importance.

It has been well documented that SARS-CoV-1, MERS-CoV, and SARS-CoV-2 viruses interact with the host angiotensin converting enzyme 2 (ACE2) through its surface spike (S) protein to commence the infection. In general, S proteins of the SARS-CoV-2 virus have two specific regions namely S_1_ and S_2_. The receptor binding domain (RBD) of the S_1_ subunit is more specific in the commencement of viral infection, as it interacts with ACE2 receptor present in the host cell. The S_2_ subunit of SARS-CoV-2 virus interacts with the host cell membrane and subsequently, transfers the genetic material to the host cell with the help of host cell proteases^6,7^. Given the reduction in effectiveness of vaccines for COVID-19 over time, antivirals can provide a suitable alternative, including novel compounds delivered via a nasal spray. In such a case, a potent entry inhibitor molecule may be useful as a therapeutic moiety for the immediate treatment of COVID-19 infection as compared to a vaccine which generally requires initial training of immune cells to provide long lasting protection^8^.

Aptamers are short oligonucleotide sequences (20–80 nucleotides) used for various biomedical applications, especially for diagnostic purposes due to their ability to fold into a three-dimensional conformation for specific binding with a suitable target^9^. Among DNA and RNA aptamers used for various applications, DNA aptamers are preferred due to their stability over RNA aptamers as they lack a hydroxyl group at the 2’-position. Moreover, numerous attractive features of DNA aptamers as compared to antibodies, e.g., low immunogenicity, amenable to surface modification, economical, and less stringent storage conditions, make them an excellent tool for biosensing and therapeutic applications^10^. Systematic evolution of ligands by exponential enrichment technique (SELEX) is a conventional technique used for isolation of the most suitably fit aptamer molecules from a library of randomly synthesized aptamers (∼ 10^15^–10^17^ molecules) using an artificially synthesized or naturally available target^11^. This process includes repetitive steps of target binding with increasing stringency in each cycle and the removal of unbound single-stranded aptamers followed by elution and amplification through polymerase chain reaction (PCR).

**Figure 1:**
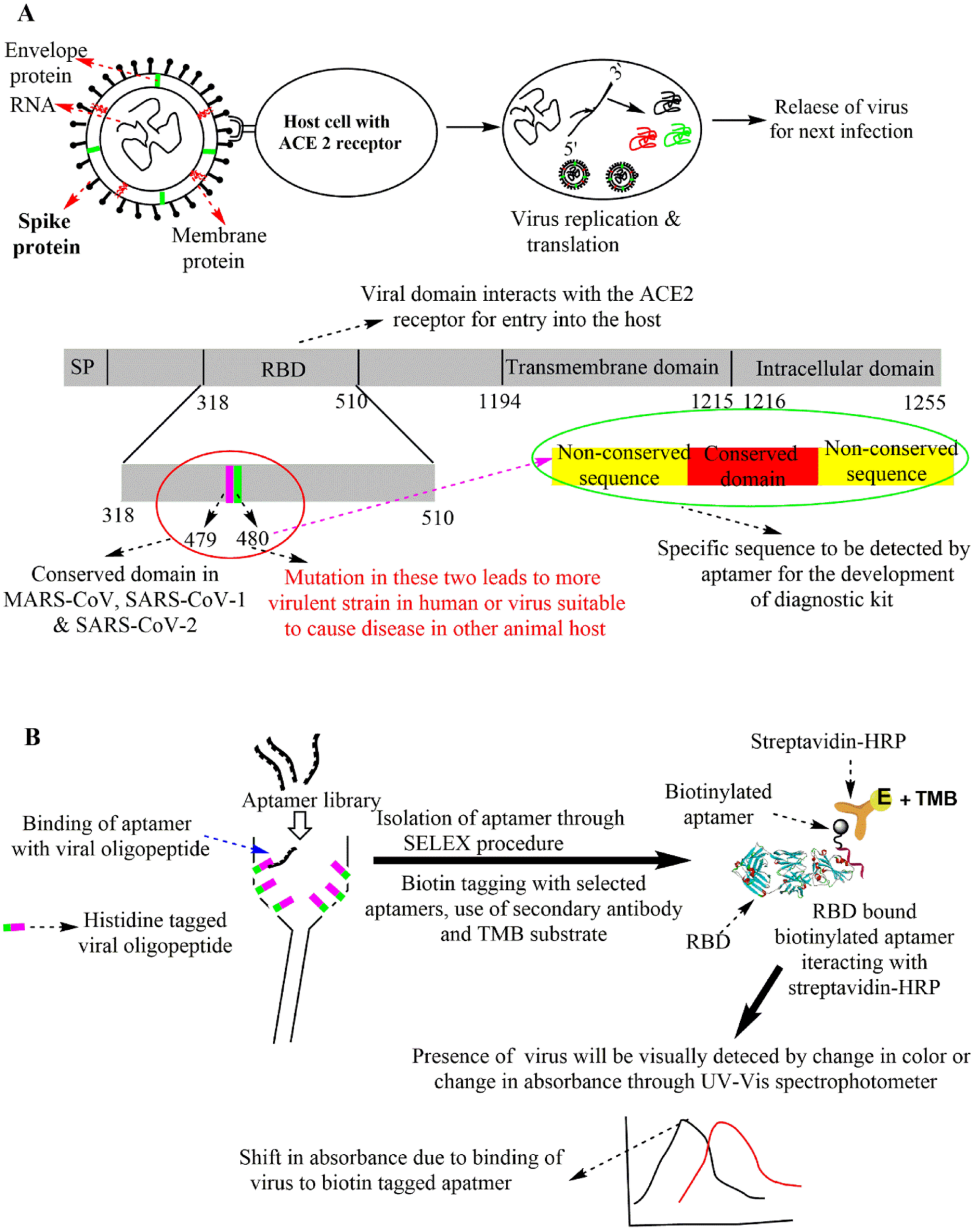
(**A**) Scheme showing selection of suitable viral oligopeptide sequence as a suitable target. (**B**) Schematic representation of isolation and identification of aptamers using SELEX.

A scientific report by Qu et al. discussed the importance of two amino acids positioned at 479 and 487 in the RBD region as an inevitable region for progression and host preference for transmission of this disease^12^. Based on these literature reports, we carried a preliminary bioinformatic analysis of spike proteins of three coronavirus strains (MERS-CoV (AKN11074.1)^13^, SARS-CoV-1 (AAT74874.1)^14^ and SARS-CoV-2 (QII57161.1)^15^) to select a suitable viral oligopeptide target for the development of a diagnostic kit. Analysis of the sequence of all three coronaviruses revealed the presence of multiple conserved domains in the spike protein. The initial bioinformatic analysis allowed us to select a viral oligopeptide sequence with a variable region (VR) flanked on either side of the conserved region (CR) as a suitable target (Fig. 1a) for the isolation and identification of an aptamer using SELEX (as depicted in Fig. 1b). During the screening, five aptamers were identified, and their binding potentials were examined through in vitro experiments using purified RBD and in silico tools (docking and molecular dynamics). Encouraged by similar results of the in vitro binding and in silico experiments, the diagnostic potential of the selected aptamers was checked using SARS-CoV-2 virus. It was hypothesized that molecules possessing excellent diagnostic potential may also have therapeutic potential given their ability to recognize and bind to the RBD. As such, antiviral efficacy was checked against the SARS-CoV-2 virus and, subsequently, the specificity of aptamers towards RBD was also analyzed in a pseudoviral system expressing ACE2 receptors^16^.

## Materials and methods

### Aptamers and reagents

Histidine-tagged viral oligopeptides were obtained from GenScript, USA. Aptamer library (5′-GGT ATT GAG GGT CGC ATC N(40) GA TGG CTC TAA CTC TCC TCT-3′), COV-Fwd (5̍-Bio/GGT ATT GAG GGT CGC ATC-3′) and COV-Rev (5′-AGA GGA GAG TTA GAG CCA TC-3′) were obtained from Integrated DNA Technologies (IDT) Belgium. His select Nickel Magnetic agarose bead (Magnetic Ni–NTA slurry) was purchased from Sigma and Streptavidin Mag Sepharose was purchased from Merck, India. PCR buffer, dNTP, Taq pol were purchased from Takara Taq™. Genelute Gel Extraction Kit, Sodium phosphate, Sodium chloride, Imidazole, Tris–HCl, Urea, SDS, Xa/LIC Cloning Kit, Dulbecco's Phosphate Buffered Saline (DPBS), Bovine serum albumin (BSA), 3,3′,5,5′-Tetramethylbenzidine (TMB), Streptavidin horseradish peroxidase were purchased from Sigma Aldrich. Agarose and DNase free water were purchased from Invitrogen. Spike Glycoprotein Receptor Binding Domain (RBD) from SARS-Related Coronavirus 2, Wuhan-Hu-1 with C-Terminal Histidine Tag, Recombinant from HEK293 Cells (Cat No. NR-53800) was obtained as a gift sample from BEI resources, USA.

### Selection and synthesis of viral oligopeptide target

Selection of a suitable viral oligopeptide is a vital aspect for the screening of appropriate aptamers in the development of a diagnostic kit. Factors like, sequence and length of viral oligopeptide can also play a very important role in the successful development of a diagnostic kit. In addition, the selected sequence of viral oligopeptide must be stable in the in vitro conditions and mimic the conformation of viral surface protein (spike protein of the SARS-CoV-2). Thus, for the selection of suitable viral oligopeptide, multiple sequence alignment of spike proteins of three viral strains (MERS-CoV (AKN11074.1), SARS-CoV-1 (AAT74874.1) & SARS-CoV-2 (QII57161.1)) were performed. Based on the sequence analysis, viral oligopeptide target was chosen and later artificially synthesized from GenScript, USA.

### Isolation and identification of specific aptamer for the selected target

#### Preparation of histidine-tagged viral oligopeptide and activation of aptamer library

The histidine-tagged viral oligopeptide target was prepared by incubating the magnetic Ni–NTA slurry with suitable viral oligopeptide in a 25 µl binding buffer solution (20 mM Sodium dihydrogen phosphate, 500 mM Sodium chloride, 5 mM Imidazole, pH 7.4). The mixture of viral oligopeptide and magnetic Ni–NTA slurry was incubated at 4 °C for 3 h and unbound viral oligopeptide was washed after the application of a magnet. Folding of the aptamer library is a very important step to avoid self-annealing and it was achieved by incubating the desired concentration of aptamer in a binding buffer at 90 °C for 10 min and immediately transferred to the ice for 30 min. The starting aptamer library was 78-mer oligonucleotide sequence in which two defined primer regions flanked a central region comprising of 40 nucleotides. The activation of aptamers was performed before the commencement of each round of SELEX.

#### SELEX (negative and positive selection)

To screen and identify suitable aptamers that can bind to the selected viral oligopeptide target, ten rounds of SELEX were performed. Starting with a random aptamer library may have a certain affinity for solid support (magnetic Ni–NTA slurry) so, as a strategy of negative selection, aptamer (10 µM) was incubated for 30 min with magnetic Ni–NTA slurry alone. Unbound aptamers were collected and further incubated with histidine-tagged viral oligopeptide for 1 h during the positive selection process. The amount of viral oligopeptide coated on magnetic Ni–NTA slurry was kept constant for two rounds of SELEX and subsequently, the amount taken was gradually decreased. The amount taken up to the next ten rounds of SELEX was 50, 25, 12.5 and 6.25 µg, respectively. After incubation of aptamer with viral oligopeptide, the aptamer-oligopeptide conjugate was eluted with elution buffer (20 mM Sodium dihydrogen phosphate, 500 mM Sodium chloride, 500 mM Imidazole, pH 7.4). Eluted fraction (aptamer-oligopeptide conjugate) was purified using Genelute Gel Extraction Kit. To obtain aptamers for the subsequent round of SELEX, purified eluate DNA aptamer was amplified using Taq DNA Polymerase (5 U/µl), 4 mM MgCl_2_, 2 µM each of Fwd and Rev primers, and 800 µM dNTPs. The amplification of aptamer was performed in a thermal cycler (Eppendorf) with the following cycle conditions: 95 °C for 15 min followed by 30 cycles of 95 °C for 30 s, 50 °C for 20 s, and 72 °C for 30 s. PCR products were visualized in 1.5% agarose gel and again purified by Genelute Gel Extraction Kit. Finally, a single-stranded aptamer was obtained from the double-stranded PCR product using a Streptavidin Mag Sepharose kit (according to the manufacturer’s protocol) for the subsequent rounds of SELEX^17^.

#### Cloning and sequencing of the selected aptamer

To analyze the sequence of the screened aptamers, eluted aptamers after 10 rounds of SELEX were amplified using non-biotinylated forward and reverse primer and finally purified by Genelute Gel Extraction Kit. The purified double-stranded PCR product was cloned in pET-32 Xa/LIC vector using the ligation-independent cloning (LIC) technique. More precisely, the complementary overhang of the PCR product (amplified screened aptamer after 10 rounds of SELEX) was produced by incubating it with T4 DNA polymerase at 22 °C for 30 min (according to the manufacturer’s instruction). Finally, the annealed recombinant product was obtained by incubating the linearized pET-32 Xa/LIC vector with screened aptamers at 22 °C for 5 min. Then, ligated aptamer-pET-32 Xa/LIC recombinant product was transformed into *E. coli* DH5α competent cells and plated on SOC agar medium containing ampicillin (40 µg/ml). The plated cells were incubated overnight to check the positively transformed colonies. Finally, recombinant plasmids isolated from different transformed colonies were sent to Eurofins, India for sequence analysis of the screened aptamers^18^.

#### Synthesis of biotin-tagged aptamer

During the sequence analysis of screened aptamers, five novel aptamers were identified. Then, biotin labelled aptamers (Apt@R_2_, Apt@T_2_, Apt@S_2_, Apt@U_2_ & Apt@W_2_) were synthesized from Eurofins, India. Further, these biotins labelled aptamers were used to check their diagnostic potential by using SARS-CoV-2 virus.

### Binding efficacy of selected aptamers

#### In vitro binding assay using RBD

The diagnostic potential and binding efficacy of the screened aptamers were assessed using RBD protein (BEI Resources, USA). In this assay, initially, RBD conjugated Ni–NTA (Ni–NTA@RBD) was prepared by incubating histidine-tagged RBD of SARS-CoV-2 protein with magnetic Ni–NTA slurry at 4 °C for 3 h in ELISA plate. The unbound RBD was washed thoroughly after the application of a magnet. The nonspecific sites of the ELISA well and magnetic Ni–NTA slurry were blocked by adding 300 μl of 1% BSA and incubated at 37 °C for 1 h. Then, unbound BSA was removed by washing thrice and the plate was dried at 37 °C for 10 min. Activated aptamer solution (0.3 μM, 50 μl) was added to Ni–NTA@RBD and incubated at 37 °C for 2 h. Subsequently, wells were washed three times with phosphate-buffered saline (PBS) and biotinylated aptamers were detected by incubating 100 μl streptavidin-conjugated horseradish peroxidase (SA-HRP) at 37 °C for 10 min. After incubation, the wells were washed three times with PBS under mild shaking and 100 µl TMB solution (substrate) was added to each well and incubated at room temperature in dark. In the assay, the enzymatic reaction was conducted for 30 min, and then stopped by adding 50 µl 1 M HCl. Finally, absorbance was measured at 450 nm using a microplate reader (Thermofischer, Finland) and a spectrum was taken in the wavelength range of 200–800 nm using UV–Visible spectrophotometer (Shimadzu, Japan)^17^.

#### In vitro binding assay using SARS-CoV-2 virus

In vitro binding assay of biotin labelled aptamers using SARS-CoV-2 viral strain was performed at Institute of Life Sciences (ILS), Bhubaneswar. SARS-CoV-2 virus (∼10^6^) was coated in 96 well ELISA plates and incubated at 4 °C overnight. The next day, wells were washed twice with PBS and nonspecific sites were blocked with 300 μl 1% BSA and incubated at 37 °C for 1 h. Subsequently, the unbound BSA was removed by washing thrice with phosphate-buffered saline supplemented with 0.1% Tween 20 (PBS-T) and then, the ELISA plate was kept for drying at 37 °C for 10 min. A total of 50 μl aptamer solution (5, 10 and 15 μl aptamer solution of 0.3 μM diluted up to 50 μl in a binding buffer: 50 mM Tris–Cl, pH 7.4, 5 mM KCl, 100 mM NaCl, and 1 mM MgCl_2_) was added to 96 well ELISA plates coated with SARS-CoV-2 virus and incubated for 2 h at 37 °C. Unbound aptamers were removed by washing three times with PBS-T. Then, 100 μl SA − HRP solution (1:1000) was added and incubated at 37 °C for 20 min. Further, the unbound antibody was removed by washing thrice with PBS-T followed by the addition of 100 µl TMB substrate and again incubated at 37 °C for 10 min. The enzyme–substrate reaction was stopped by adding a stop solution (1 M HCl). Then absorbance was measured at 450 nm in a microplate reader. Chikungunya virus was used to check the specificity of aptamers and 1X PBS taken as a negative control^17^.

### Antiviral activity using SARS-COV-2 virus

#### Cell line and virus

The Vero cell line was procured from NCCS, Pune, India. Cells were maintained in Dulbecco’s modified Eagle’s medium, (DMEM, PAN Biotech, Aiden Bach, Germany) supplemented with 10% Fetal bovine serum (FBS) (PAN Biotech, Aiden Bach, Germany), Gentamycin, and Penicillin–streptomycin. ILS-03 (Accession no. EPI_ISL_1196305) strain of SARS-CoV-2 was successfully isolated and adapted in Vero E6 cells at ILS, Bhubaneswar and this strain was used for this study^19,20^.

#### Cytotoxicity assay

3-(4,5-dimethylthiazol-2-yl)-2,5-diphenyltetrazolium bromide (MTT) assay was performed to determine the cytotoxicity of Aptamer R, T, J, and L using EZcount™ MTT cell assay kit (Himedia, Mumbai, India) in Vero cells as mentioned earlier^21^. In brief, Vero cells were seeded in a 96 well plate at the density of approximately 10,000 cells per well. After reaching 80% confluency, cells were treated with different concentrations of aptamer R and T (1, 2, 3, and, 4 µM) and J and L (2, 4, 6, and, 8 µM) in triplicate form. 1X PBS was taken as a reagent control. After 22 h, plate was washed with 1X PBS and subsequently, 100 μl MTT reagent (1 mg/ml diluted in DMEM serum free media) (Himedia, Mumbai, India) was added to the cells and incubated for 2–3 h at 37 °C. Next, the media was removed and 100 μl of solubilization buffer was added followed by incubation at 37 °C for 15 min to dissolve the formazan crystals. Finally, the absorbance was measured at 570 nm using a multimode plate reader and the metabolically active cell percentage was compared with the control cells to determine the cytotoxicity^21^.

#### Viral infection

At 80% confluency, vero cells were infected with SARS-CoV-2 at 0.1 multiplicity of infection (MOI) along with a maximum non-cytotoxic concentration of different aptamer compounds (R, T, J, and L) followed by 90 min incubation with shaking at every 10 to 15 min interval. After 90 min of incubation, the cells were washed twice with 1X PBS and incubated at 37 °C after the addition of DMEM complete media. Supernatants were harvested at 22 hpi (hour post-infection) and used for RNA extraction.

#### RNA extraction and qRT-PCR

To determine the viral copy number and anti-viral effect, qRT-PCR was performed according to a previously described method^19,20^. For this, viral RNA was isolated from the supernatants using the TAN Bead Maelstrom 4800 automated RNA extraction platform as per the manufacturer’s instructions and cDNA was synthesized using random hexamers by the Prime Script First strand cDNA synthesis kit (Takara, Kusatsu, Japan). The synthesized cDNA was used as a template to amplify the Nucleocapsid (N) genes of SARS-CoV-2 using specific primers (NCFP: GTAACACAAGCTTTCGGCAG and NCRP: GTGTGACTTCCATGCCAATG) by qRT-PCR (Mesagreen SYBR Green-No ROX, Eurogentec, Belgium). The viral copy number was determined for the above-mentioned samples by generating the standard curve of the SARS-CoV-2 N gene. The percentage of copy number/ml was calculated from the corresponding Ct values of all the samples^22^.

### Pseudovirus based entry assay to determine the effect of aptamers upon SARS-CoV-2 entry

#### Cell line

Human embryonic kidney (HEK) 293 T cells (#CRL-3216) (obtained as a gift sample from University of Wisconsin-Madison, USA) were maintained in DMEM supplemented with 10% fetal bovine serum (FBS) along with penicillin and streptomycin antibiotics (Gibco) at 37 °C and in 5% CO_2_.

#### Plasmids

Plasmid (pHDM-IDTSpike-fixK) encoding codon optimized SARS-CoV-2 spike protein (S) glycoprotein (Genbank NC_045512) was obtained from BEI Resources (BEI # NR-52514). The lentiviral backbone plasmid (pHAGE2-EF1aInt-ACE2-WT) expressing the human ACE2 gene (BEI # NR-52512) was used for overexpression studies. Lentiviral plasmid (pHAGE-CMV-Luc2-IRES-ZsGreen-W) integrated with the luciferase as well as ZsGreen ORFs (BEI # NR-52516) was used in this study. Lentiviral plasmids encoding the HIV helper proteins including Gag-Pol (pHDM-Hgpm2) (BEI # NR-52517), Tat (pHDM-tat1b) (BEI # NR-52518) and Rev (pRC-CMV-Rev1b) (BEI # NR-52519) were used as indicated. Plasmids expressing the Vesicular Stomatitis Virus (VSV) glycoprotein (G) (Addgene#138479) was obtained from Addgene (MA, USA).

#### Production of SARS-CoV-2 based pseudoviruses

Pseudotyping HIV-1 based lentiviral system to produce reporter viral particles expressing SARS-CoV-2 spike glycoproteins was originally described by Crawford et al.^23^. Briefly, it involves co-transfecting HEK 293 T cells with a lentiviral plasmid expressing the firefly luciferase protein, plasmids encoding HIV-1 proteins- Gag-Pol, Rev and Tat which are necessary for the virion formation, and plasmid encoding either SARS-CoV-2 S, VSV-G, or empty vector by using calcium phosphate transfection kit (Takara-Bio). The supernatants containing the viral particles were harvested at 60 h post-transfection and centrifuged at 1000×*g* for 10 min to remove any cell debris. Reporter pseudoviruses thus produced were used to infect either HEK293T cells or HEK293T cells overexpressing human hACE2 (HEK293T-ACE2).

#### Pseudoviral entry assay

Pseudovirus entry assay was performed as described previously by Das Jana et al.^24^. HEK 293 T cells were transduced with lentiviral plasmids to express hACE2 protein. At 24 h, the cells were infected with lentiviruses pseudotyped with either SARS-CoV-2 S or VSV G at its surface. The efficiency of these pseudoviruses to enter HEK293T-ACE2 was quantified by measuring the luciferase activity within the infected cells at 18 hpi. The cells were lysed and a luciferase assay was performed using the luciferase assay reagent (E1501, Promega, USA). The luciferase unit thus generated was measured by using a luminometer -Glomax 20/20 (Promega).

#### Aptamers on pseudovirus entry

Lentiviruses pseudotyped with either SARS-CoV-2 S or VSV G were treated with different concentrations of Aptamers (R, J, T and L) for 30 min at RT. HEK 293 T cells overexpressing hACE2 were infected with respective aptamer treated viral inoculum at 37 °C. Infected cells were harvested at 18 hpi and a luciferase assay was performed. The amount of virus entering the cells was quantified by a luciferase unit measured using a luminometer -Glomax 20/20 (Promega, USA). Each experiment was performed thrice and each data is representative of three independent experiments.

#### MTT assay

The toxicity of the aptamers (if any) in cells was determined by MTT assay as described previously by Mosmann (1983)^25^. Briefly, HEK 293 T cells were seeded in a 96-well plate at a density of 40,000 cells per well. Thereafter, the cells were treated with different concentrations of the Aptamers (R, J, T and L) for 24 h at 37 °C in 5% CO_2_. The media was removed, followed by the addition of 100 μl of MTT reagent (5 mg/ml, SRL) in PBS to the cells and incubated for 3 h at 37 °C. Formazan crystals thus formed in each well were resuspended in 100 μl of dimethyl sulfoxide (DMSO) (Sigma, USA). The absorbance was measured at 595 nm using an Epoch 2 microplate reader (BioTek Instruments). All experiments were performed in triplicate, and the cell viability was calculated as the ratio of each experimental condition to the control solvent on the same plate.

### In silico predictions of binding interactions between aptamers and RBD

#### Computational system preparation

In silico interaction studies were carried out on the experimentally determined aptamers R and J to better understand their diagnostic and antiviral potential. The secondary structures of aptamers R and J were predicted from their sequence using the Mfold web server, which calculated their minimum free energy structure^26^. The predicted secondary structure was exported in VIENNA format to the RNACOMPOSER webserver^27^. It was then used to build a 3D model for the R and J aptamers and the output file was saved in a pdb format. The predicted 3D structure of the R and J aptamers were subjected to molecular dynamics (MD) simulations for 200 ns (as described in the section below) and the refined lowest energy structure determined from the 200 ns trajectory was used as an input file for the molecular docking studies.

#### Molecular docking

To examine the potential binding of the aptamers to the RBD of the SARS-CoV-2 spike protein (S1), the lowest energy structure of aptamers R and J were docked against the crystal structure of SARS-CoV-2 (PDB ID: 6M0J)^28^ using the HDOCK^29^ webserver. This crystal structure of SARS-CoV-2 S1 protein is co-crystallized with ACE2 thus making a S1-RBD-ACE2 complex. As the in vitro experiments determining the binding of aptamers were carried out using the RBD only, the ACE2 receptor was deleted. All default docking protocols were applied to obtain the computed binding poses of the R and J aptamers to S1-RBD.

#### Molecular dynamics

The R and J aptamer-S1-RBD complexes were then subjected to MD simulations. The protein aptamer complex was solvated explicitly using a TIP3P^30^ orthorhombic water box that extended 10 Å away from the system and the overall charge was neutralized by adding a suitable number of counter ions. The DNA-OL15^31^ and ff14SB^32^ force fields were used to generate the aptamer and protein topologies, respectively, and MD simulations were carried out using the GPU-enabled Amber18 pmemd engine. In the first step of MD simulation, only the water molecules and counter ions were minimized using the conjugate gradient (CG) method for 500 steps, followed by 10,000 steps of CG minimization for the entire system. Thereafter, the system was gradually heated from 0 to 300 K using a constant NVT ensemble over 50 ps with a Berendsen thermostat and temperature coupling value of 2.8 ps. To correct the density of the system, a 500 ps simulation was performed using a constant NPT ensemble at 300 K and 1 atm with the temperature and pressure coupling values set to 2.0 ps. The system was then switched back to the NVT ensemble and further equilibrated for 500 ps. Following the minimization and equilibration phase, a 200 ns NVT production run was collected. Long-range electrostatics were accounted for by using particle mesh Ewald, all covalent bonds involving hydrogen atoms were constrained with the SHAKE algorithm, periodic boundary conditions were enforced using a nonbonded cut-off distance of 12 Å, and a time step of 1.0 fs was utilized. Analysis was performed with the cpptraj and ptraj programs available in the AmberTools18 suite^33,34^. Root-mean-square-deviations (RMSDs) and root-mean-square-fluctuations (RMSFs)^35^ monitored the structural stability and motions of the proteins for each simulation. RMSD values of the backbone protein atoms are provided in Figs. S1–S3.

#### Cluster analysis

Cluster analysis is a method of determining a population ensemble during MD simulation by grouping similar conformations together over the trajectory. In this study, the cpptraj “average-linkage” algorithm was used,^33,36^ where for the distance metric, RMSD of atoms with a sieve of 10 was applied.

### Statistical analysis

Data obtained through in vitro studies were statistically analyzed either via one-way analysis of variance (ANOVA)- Bonferroni post-test or a two-way ANOVA-Bonferroni post-test using Graph Pad Prism and values denoted by ** (p < 0.01) and *** (p < 0.001); n = 3, were considered significant.

## Results and discussion

### Selection criteria of viral oligopeptide target for SELEX

**Figure 2:**
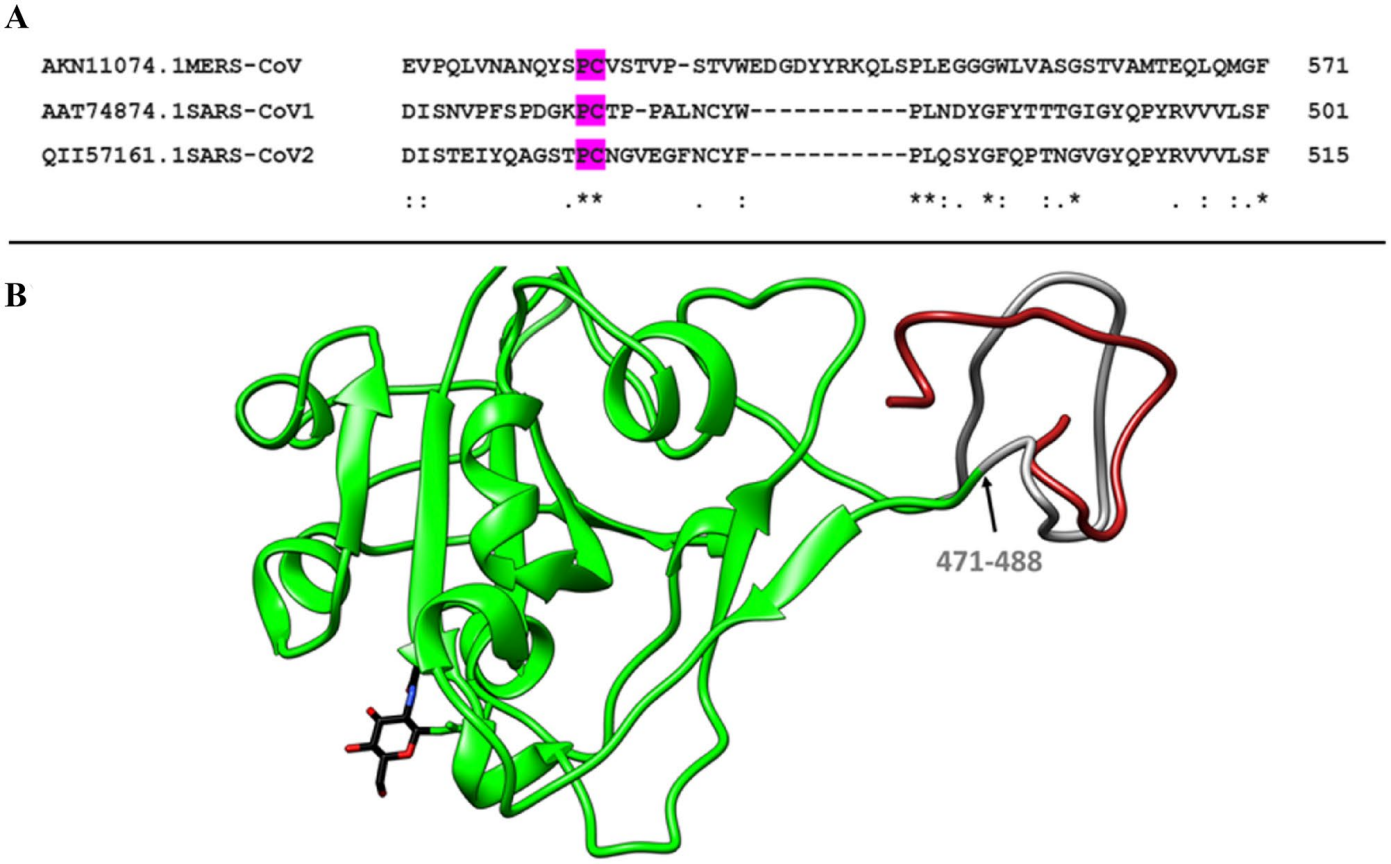
(**A**) Multiple sequence alignment data of spike protein of MERS-CoV, SARS-CoV-1 and SARS-CoV-2. (**B**) Comparison between the SARS-CoV-2 S1-RBD domain (green) conserved region of residues 471–488 (grey) in the native structure selected for SELEX study with last trajectory frame obtained from 300 ns of molecular dynamic simulations of the oligopeptide (red).

This study aimed to design an aptamer that could bind to SARS-CoV-2 with high specificity. Factors like the sequence and length of viral oligopeptide can play a very important role in the development of a diagnostic kit. In addition, the selected sequence of viral oligopeptide must be stable under in vitro conditions and needs to mimic the conformation of the viral surface protein (spike protein of the SARS-CoV-2 virus). Thus, in this process of designing a suitable viral oligopeptide, multiple sequence alignment of spike proteins of three viral strains (MERS-CoV (AKN11074.1), SARS-CoV-1 (AAT74874.1) and SARS-CoV-2 (QII57161.1)) were performed. Based on the sequence analysis of residues ranging from 310 to 510, residues 479 and 480 located in the RBD region of the SARS-CoV-2 virus were selected as the conserved region (CR) (Fig. 2A). Further, two variable regions (VRs) of eight amino acids residues were selected on either side of CR. Thus, 18 amino acid long histidine-tagged viral oligopeptide (EIYQAGSTPCNGVEGFNCHHHHHH) was synthesized from Genscript, USA, for the identification of suitable aptamers using the SELEX procedure. It is well-known that the secondary native conformation of any protein is generally stabilized by the number of α helices and the β pleated sheets^37,38^. Mimicking such secondary structures of designed oligopeptides during in vitro conditions with the native protein under study is a very important criteria for suitable selection and isolation of a specific aptamer using the SELEX technique. To analyze the secondary structure information of the chosen viral oligopeptide under study, circular dichroism (CD) spectroscopy (Instrument name-JASCO CD, Model name-J-1500, Sl. No- A022361638) has been performed. CD spectroscopy data reveals the percentage of helix, β sheets, turn and random coils as 17.6, 62.6, 6 and 13.9%, respectively (Fig. S8). Further, to analyze the selected synthetic viral oligopeptide target (combination of CR and VRs with a total of eighteen amino acids in length) in detailed manner, molecular dynamics simulations have been performed. Molecular dynamics simulations of the oligopeptide performed here confirmed retention of the native structure of the SARS-CoV-2 S1-RBD reported in the crystal structure (Fig. 2B). However, CD spectroscopic data is not in well agreement with molecular dynamics simulations studies which may be attributed to the fact that CD spectroscopy has been performed for the selected oligopeptide as an independent moiety rather than integral part of SARS-CoV-2 S1-RBD.

### Screening of DNA-aptamers using SELEX and sequence analysis

SELEX is a conventional technique used for the isolation of the most fit aptamer molecules. From a pool of randomly synthesized aptamers (∼10^17^ molecules), suitable aptamers were screened using ten rounds of SELEX. Further, positive and negative selections were performed to obtain highly specific aptamers for the desired target under study. The obtained aptamers were amplified and subsequently converted into a single-stranded form, which was used for the next round of SELEX. After ten rounds of SELEX, finally, the PCR purified screened aptamers were cloned into a pET-32 Xa/LIC vector and sequence of screened aptamers were analyzed. The sequence of these screened aptamers with primer region (R, S, T, U, and W) and aptamers without primer region (J, K, L, M, and O) has been summarized in Table 1. In order to analyze the diagnostic potential, the aptamers (R, S, T, U, and W) were biotinylated and color was produced using streptavidin-conjugated horseradish peroxidase (SA-HRP) in the presence of TMB substrate. Further, from the screened aptamers, R, S, T, U, and W aptamers have been selected for diagnostic potential studies because these were obtained inherently during SELEX, whereas aptamers R, T, J, and L have been used to determine their antiviral potentials because these aptamers have shown the best results during in vitro binding experiments using the SARS-CoV-2 virus.

**Table 1:** Screened aptamer sequences obtained through SELEX using viral oligopeptide.

S. No	Sample code	Aptamer sequences flanked with and without primer region
1	R	**GGTATTGAGGGTCGCATC**GTTACTGGACCAAACAGCACAAAGGACGCCCATAAACCCG*GATGGCTCTAACTCTCCTCT*
2	S	**GGTATTGAGGGTCGCATC**GAAACAGGGCGTGATACGACGCGACGGCGTACTGTGGTGG*GATGGCTCTAACTCTCCTCT*
3	T	**GGTATTGAGGGTCGCATC**GTAATAGCGCATGTTGTGAGGCAGGCGCGAATCAGCTCGTGA*GATGGCTCTAACTCTCCTCT*
4	U	**GGTATTGAGGGTCGCATC**GTAATAGCGCATGTTGTGAGGCGGCGGAGTCATGTCGTGG*GATGGCTCTAACTCTCCTCT*
5	W	**GGTATTGAGGGTCGCATC**GACACCGGGAGTGATACGTCGCGAGCACGTACTGTGTTGG*GATGGCTCTAACTCTCCTCT*
6	J	GTTACTGGACCAAACAGCACAAAGGACGCCCATAAACCCG
7	K	GAAACAGGGCGTGATACGACGCGACGGCGTACTGTGGTGG
8	L	GTAATAGCGCATGTTGTGAGGCAGGCGCGAATCAGCTCGTGA
9	M	GTAATAGCGCATGTTGTGAGGCGGCGGAGTCATGTCGTGG
10	O	GACACCGGGAGTGATACGTCGCGAGCACGTACTGTGTTGG

**Bold and italics represent the forward primer and reverse primer region present in the screened aptamer, respectively.

### Aptamer binding for RBD using in vitro binding assay

**Figure 3:**
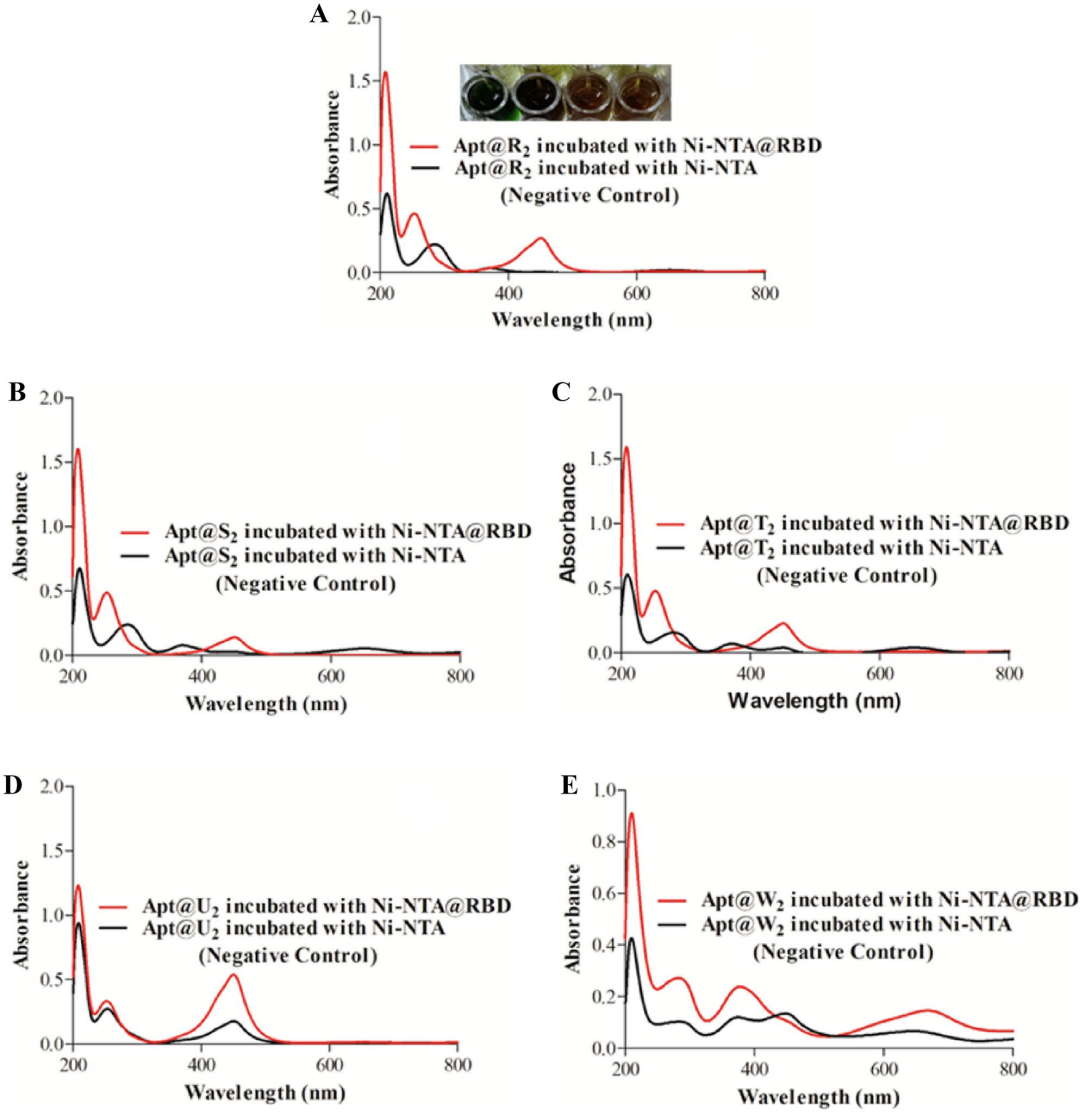
UV–Visible spectra of screened aptamers with RBD along with its negative control; (**A**) Aptamer R incubated with Ni–NTA@RBD; (**B**) Aptamer S incubated with Ni–NTA@RBD; (**C**) Aptamer T incubated with Ni–NTA@RBD; (**D**) Aptamer U incubated with Ni–NTA@RBD; (**E**) Aptamer W incubated with Ni–NTA@RBD, along with subset figure showing ELISA result with change in color (yellow to green).

To analyze the diagnostic potential of the screened aptamers (R, S, T, U and W), the binding efficacy of these aptamers were first tested using purified RBD. A histidine-tagged RBD was immobilized on magnetic Ni–NTA beads and then biotin-tagged aptamer samples were incubated with Ni–NTA@RBD. Finally, the binding efficacy of the screened aptamers were tested using a SA-HRP and TMB substrate. The obvious color change (yellow to green as depicted in Fig. 3A) provided confirmation that binding occurred between the aptamers and RBD. Further, the binding of screened aptamers was also analyzed with an absorbance spectra during their incubation with RBD. Here, aptamers incubated in the absence of RBD served as a negative control. During the incubation of aptamer R with Ni–NTA@RBD, two conspicuous peaks appeared at 253 and 451 nm, respectively, while during incubation of aptamer R with Ni–NTA bead only (negative control), the peak appeared at 285 nm. The two conspicuous peaks at 253 and 451 nm disappeared when the aptamer was incubated with a magnetic bead only (Fig. 3A). During aptamer S incubation with Ni–NTA, peaks appeared at 285 and 369 nm respectively while it has been shifted to 253 and 451 nm during incubation of aptamer S with Ni–NTA@RBD (Fig. 3B). Similarly, when aptamer T was incubated with Ni–NTA@RBD, two conspicuous peaks appeared at 254 and 450 nm. However, during incubation with Ni–NTA, peaks at 254 and 450 nm disappeared, and two new peaks appeared at 281 and 370 nm (Fig. 3C). During aptamer U incubation with Ni–NTA@RBD, a clear shifting in the peak was not observed but peak intensity decreased considerably during aptamer incubation with and without RBD (Fig. 3D). In the case of aptamer W, a slight shift in the absorbance peak at 378 nm was observed during incubation with Ni–NTA@RBD as compared to the negative control. A new peak also appeared at 449 nm during aptamer W incubation with Ni–NTA only while no corresponding peak appeared during aptamer incubation with the Ni–NTA@RBD system (Fig. 3E).

In summary, the binding assay of screened aptamers with RBD clearly suggest that the aptamers are efficiently binding with RBD which has been confirmed by the shifting, appearance, or disappearance of peaks during their incubation with RBD in comparison to their incubation in absence of RBD. Further, the dissociation constant (Kd) value of the aptamer R was determined using carbon dots as a fluorescent probe (Table S5). The obtained Kd value of aptamer R was found 125.26 nM.

### Aptamers R and T shows highest diagnostic potential with good binding efficacies with SARS-CoV-2

**Figure 4:**
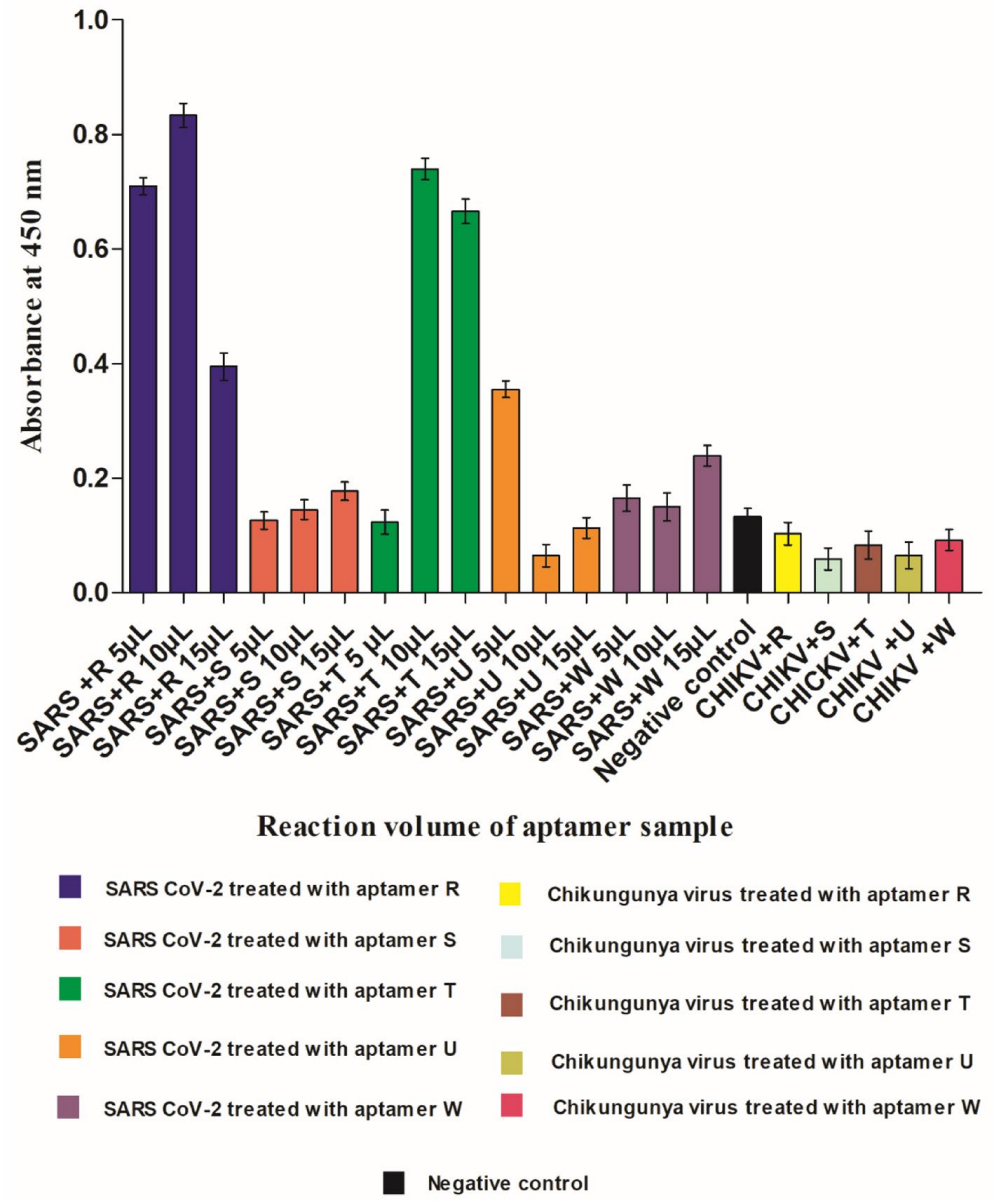
In vitro binding efficacy test of screened aptamers using SARS-CoV-2 virus. Different volumes (5, 10 and 15ul having a concentration of 0.3 μM) of all the selected aptamers (R, T, S, U and W) from the RBD binding study were taken to screen for binding efficacies using the SARS-CoV-2 virus. The bar diagram represents the absorbance at 450 nm of different aptamers.

Designing a highly selective and robust “aptamer-based detection system” for the SARS-CoV-2 virus is of paramount importance in preventing pandemic waves. Identifying potential molecules that can serve in newly designed diagnostic kits with high antiviral activity is an arduous task but can provide large potential for simultaneous detection and treatment. Due to unique features of aptamers such as small size, high stability, low immunogenicity, safety, high binding specificity and affinity towards target molecule make them a potential theranostic candidate^39,40^. Encouraged by the binding efficacy test of biotin-labeled screened aptamers (R, T, S, U, and W), their diagnostic potential was examined using the SARS-CoV-2 virus (Fig. 4). As discussed in the earlier section and shown in Fig. 3, all five selected aptamers have indicated RBD binding. Further, in an in vitro assay at three different reaction volumes (5, 10, and 15 µl having a concentration of 0.3 μM) using the SARS-CoV-2 virus, aptamers R and T showed the highest binding ability among the five screened aptamers as their corresponding absorbance at 450 nm was 0.83 and 0.74, respectively (Fig. 4). The reaction volume of 10 µl was identified to be the saturation point due to the decrease in absorbance for all five aptamers observed at 15 µl. However, the factors like the saturation of RBD at a particular viral load used or steric hindrance of the aptamer to bind with the RBD of the virus can also be attributed towards the decrease in absorbance peak at 15 µl. Additionally, the binding specificity of these aptamers were checked by taking chikungunya virus as a control. No significant absorbances were observed of aptamers in the presence of chikungunya virus (CHIKV). In summary, aptamer R and T were found to be a highly selective binder of RBD of the virus with good diagnostic potential. Hence, these can be excellent candidates for further development of theranostic agents against SARS-CoV-2.

### Thermodynamics calculations of aptamer R

In general, the function of aptamers is affected by their ability to fold and maintain a favorable binding conformation with their binding partner^41^. Thus, thermodynamically determining the folding free energy (Δ*G*_fold_) for a given aptamer sequence can provide a better understanding of aptamer stability, which is useful for predicting affinity and specificity during molecular detection and other assays^42^. The current work used the mfold server^26^ to predict Δ*G*_fold_ for the biotin-labeled screened aptamers (R, T, S, U, and W). Analyzing the Δ*G*_fold_ energy for the 5 aptamers suggested that aptamer R required higher energy to fold (approximately  − 3.5 kcal/mol) than the other aptamers which had folding energies lower than − 5 kcal/mol (Table S1). Upon closer inspection of the sequences of all 5 aptamers it was found that aptamer R had the greatest amount of A-T base pairs and the smallest amount of G-C base pairs in comparison to the other 4 aptamer sequences (T, S, U, and W) (Fig. S4, Tables S2 and S3). In addition, aptamer R that had the highest number of bases (42 out of 78) which allowed the formation of 2 helices (Fig. S5), this may suggest a greater entropic penalty for folding aptamer R relative to the other aptamers^43^.

### Aptamers showing antiviral potential against SARS-CoV-2

**Figure 5:**
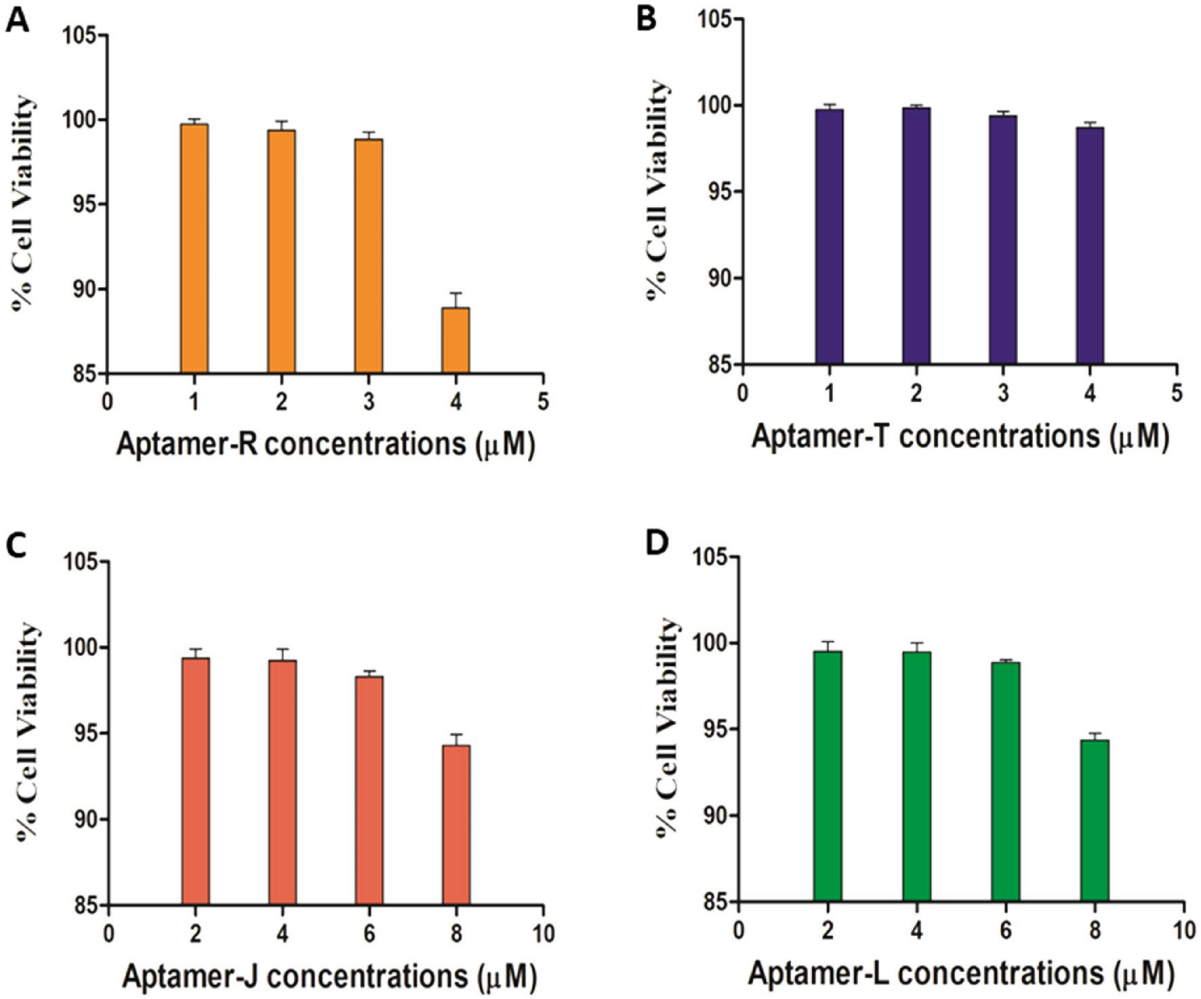
Effect of different aptamers on the viability of Vero cell: Vero cells were incubated with all the four aptamers at different concentrations for 22 h followed by addition of MTT. Absorbances were measured at 570 nm to determine the viability of cells. Bar diagram indicate the % cell viability when the cells were treated with (**A**) Aptamer R, (**B**) Aptamer T, (**C**) Aptamer J, (**D**) Aptamer L.

**Figure 6:**
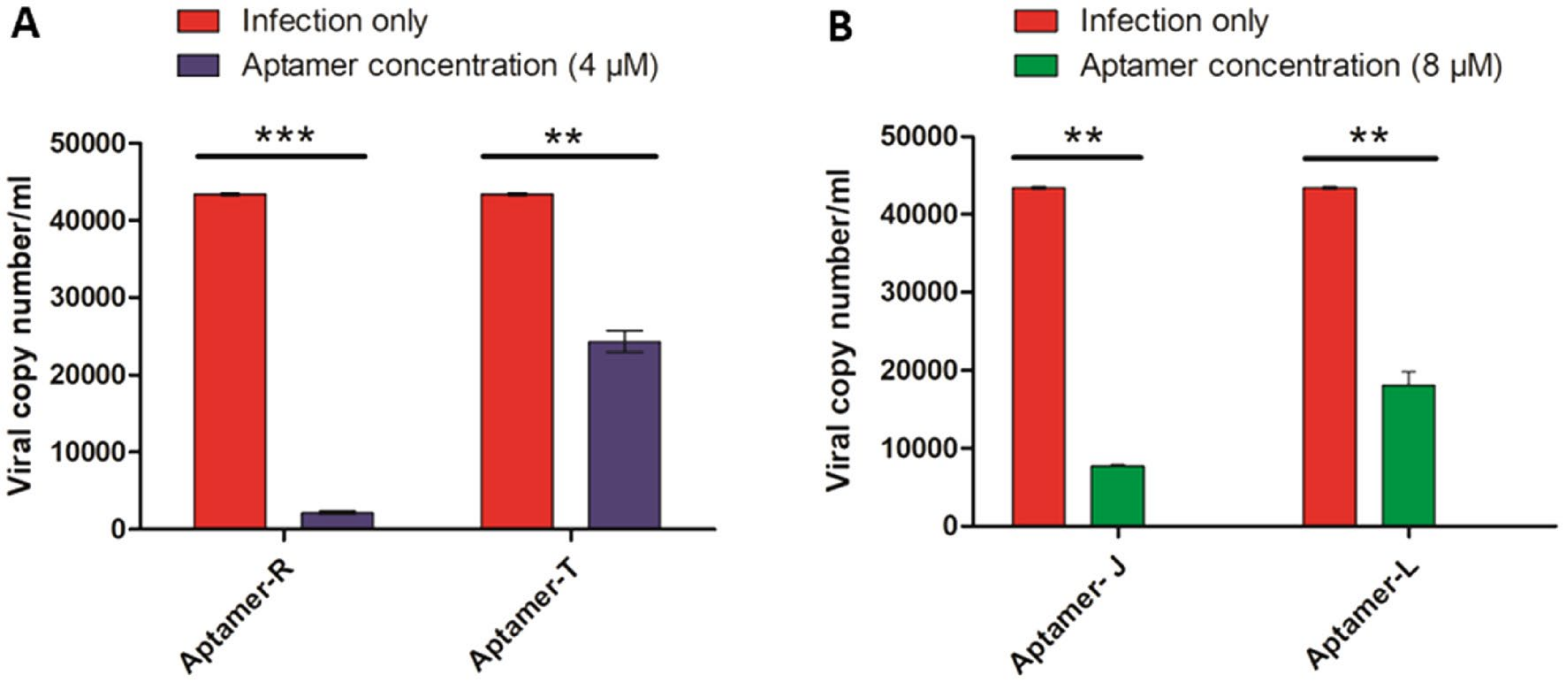
Antiviral activity of aptamers against SARS-CoV-2. Vero cells were infected with SARS-CoV-2 along with aptamers and incubated for 90 min. Supernatants were collected at 22hpi and qPCR was performed to determine the viral titer. Bar diagram indicate the viral copy number, when cells were treated with (**A**) aptamer R and T; (**B**) aptamer J and L. Statistical analysis of in vitro antiviral activity of aptamer R, T, J and L against SARS-CoV-2 was performed via two-way ANOVA- Bonferroni post-test. Significant difference was represented by ** (p < 0.01) and *** (p < 0.001); n = 3.

As, aptamer R and T (flanked with forward and reverse primer regions) showed high binding efficacy against SARS-CoV-2, thus, further antiviral studies were performed with aptamers R, T, J, and L (J and L have the same central sequence as aptamers R and T without the forward and reverse primer regions). To determine the antiviral effect of these aptamers, first the cytotoxicity was measured in the Vero cell line using MTT assay. The maximum non-toxic dose (> 90% cell viability) for aptamers R and T was found to be 4 µM while aptamers J and L showed a value of 8 µM (Fig. 5). Antiviral activity of these four aptamers were measured by qRT-PCR during the SARS-CoV-2 infection using the above-mentioned concentrations. The aptamers R and J exhibited 95.4% and 82.5% inhibition of viral infection, respectively indicating high antiviral potential against SARS-CoV-2 compared to aptamers L and T (Table 2, Fig. 6). As the aptamers were added during the infection, this reduction might be due to its interference in the viral entry stage inside the cell. Altogether, the data indicate that aptamers R and J can inhibit SARS-CoV-2 infection significantly by affecting the entry phase of the virus inside the cell.

**Table 2:** Antiviral activity of different aptamers against SARS-CoV-2.

Sample	Concentration (uM)	Ct value	Copy number/ml	% Inhibition
Infection (0.1 MOI)		25.398	43,621.18581	
Aptamer R	4	30.091	1994.156045	95.4
Aptamer T	4	26.377	22,917.9018	47.4
Aptamer J	8	28.080	7480.607116	82.5
Aptamer L	8	26.885	16,410.86434	62.3

### Aptamers inhibiting SARS-CoV-2 pseudovirus entry in cells expressing ACE2 receptors

**Figure 7:**
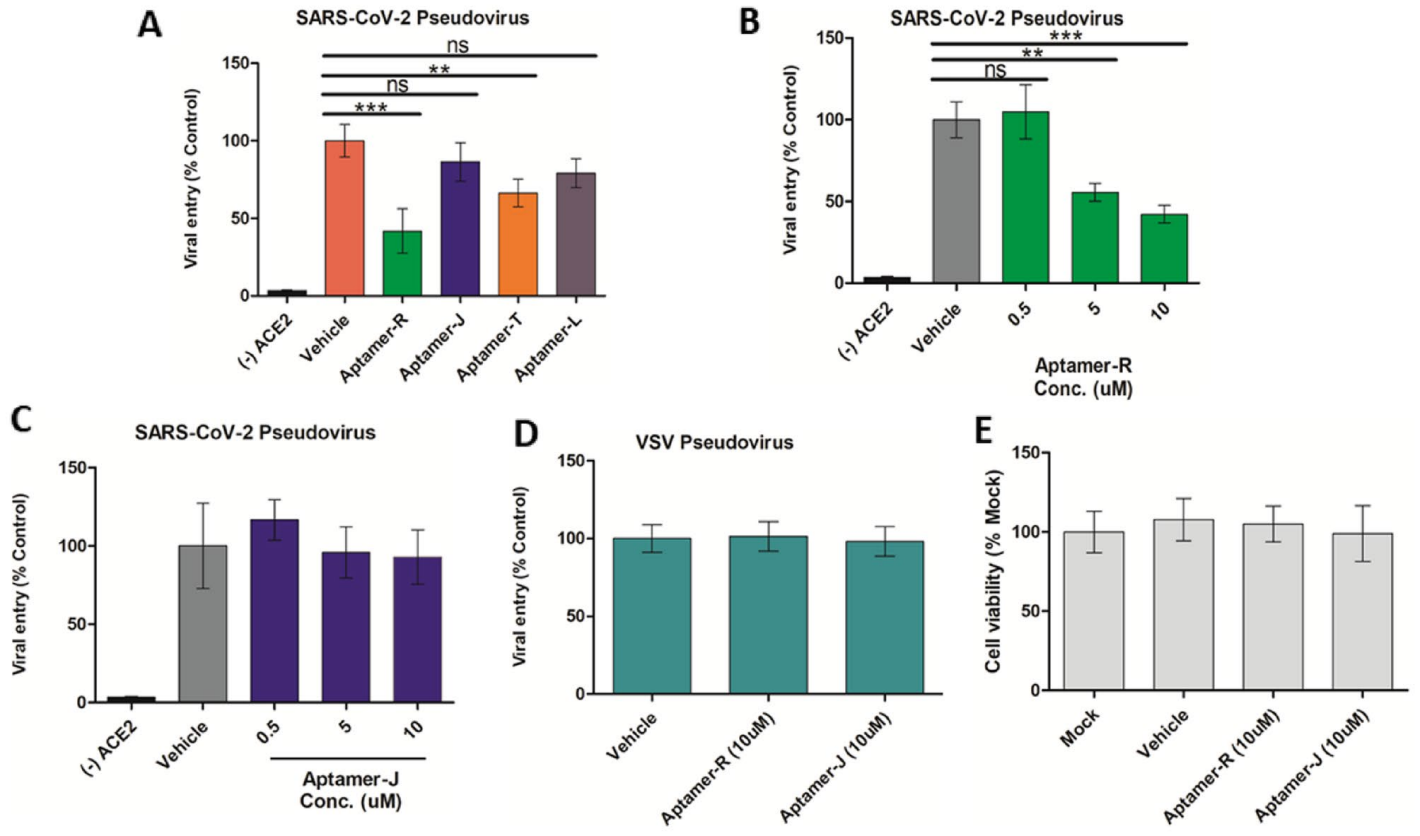
Aptamers inhibit SARS-CoV-2 S pseudotyped lentivirus entry into HEK293T-ACE2. (**A**) SARS-CoV-2 S pseudotyped reporter viruses were pretreated with vehicle control or with different Aptamers (5 uM) before infecting either HEK293T or HEK293T-ACE2 cells with the treated inoculum. Luciferase activity was measured at 18 hpi and represented as a relative percentage to the vehicle control. (**B**,**C**) SARS-CoV-2 S pseudotyped reporter viruses were treated with increasing concentrations (0.5, 5, and 10 uM) of Aptamers R and J followed by infection of the HEK293T-ACE2 cells with them. Luciferase activity was represented as a relative percentage to the vehicle control. (**D**) VSV G pseudotyped lentiviruses were pre-treated 10 uM of R and J Aptamers and used to infect HEK293T cells. (**E**) HEK293T cells were treated with different concentrations of Aptamers R and J for 24 h as indicated. Cytotoxicity of the Aptamers was determined by MTT assay. Statistical analysis of selected aptamers using pseudovirus entry assay was performed via one-way ANOVA- Bonferroni post-test. Significant difference was represented by ** (p < 0.01) and *** (p < 0.001); n = 3.

As the aptamers showed binding to the spike protein in vitro and inhibition of SARS-CoV-2 infection in Vero cells, it could be presumed that the aptamers specifically act by blocking SARS-CoV-2 entry into the host cells. Hence, we tested their efficacy for inhibiting SARS-CoV-2 entry by using a pseudovirus-based entry assay. Previous reports indicated that HIV-1-based reporter lentiviruses with the firefly luciferase ZsGreen reporter genes in their genomic backbone and SARS-CoV-2 spike protein as the sole surface glycoprotein are suitable for studying viral entry into host cells^23,44^. Therefore, we have generated reporter viruses pseudotyped with SARS-CoV-2 S and treated them with different aptamers before infecting HEK293T cells overexpressing ACE2 receptors (HEK293T-ACE2) (Fig. 7A). The efficiency of viral entry and progression of the infection into the cells was determined by measuring the luciferase gene expression at 18 hpi. As expected, SARS-CoV-2 pseudoviruses showed preferential infection towards HEK293T-ACE2 cells over the regular HEK293T cells, originating from the specificity of the spike protein for the ACE2 receptors. Treatment with 5 µM of aptamer R resulted in a 59% reduction in viral entry (Fig. 7A) while, the aptamer T and aptamer L, at the same concentration, showed 21% and 34% reductions, respectively. To our surprise, aptamer J, which contains the identical central nucleotide sequence to aptamer R, did not show any significant reduction in the viral entry as compared to antiviral activity towards live SARS-CoV-2 (Fig. 7A). However, mechanism of the antiviral activity of aptamer J needs to be further investigated.

To investigate the entry inhibitory activity of the aptamers R and J in detail, the SARS-CoV-2 pseudoviruses were pre-treated with increasing concentrations of the said aptamers, before infecting HEK293T-ACE2 cells with them. Prior incubation with aptamer R resulted in a dose-dependent decrease in pseudovirus entry while aptamer J showed no such effect (Fig. 7B,C). This data confirmed the efficacy of the aptamer R in inhibiting SARS-CoV-2 entry, possibly by direct interaction with the RBD of spike protein as evidenced in our in vitro binding assays (Figs. 3 and 4). To further confirm the specificity of this aptamer towards SARS-CoV-2 spike protein, we have generated reporter lentiviruses pseudotyped with Vesicular Stomatitis Virus (VSV) Glycoprotein (G) at their surface. As expected, the VSV G pseudotyped virus showed equal efficiency of infecting either HEK293T or HEK293T-ACE2 cells, thus confirming that the specificity towards the HEK293T-ACE2 cells is a sole attribute of the SARS-CoV-2 S pseudotyped virus. Furthermore, prior treatment of VSV G pseudotyped viruses either with aptamer R or aptamer J showed no reduction in viral entry into HEK 293 T cells (Fig. 7D). This establishes the high specificity of aptamer R in blocking the entry of SARS-CoV-2. To nullify that the inhibitory activity of aptamer R is due to its toxic effect on the cells, we performed MTT assay and tested the toxicity of the aptamers upon the HEK cells. Our data indicated that both the aptamers are non-toxic at the concentrations used for the pseudovirus reporter assay (Fig. 7E).

### Predicted binding interactions between screened aptamers and RBD

**Figure 8:**
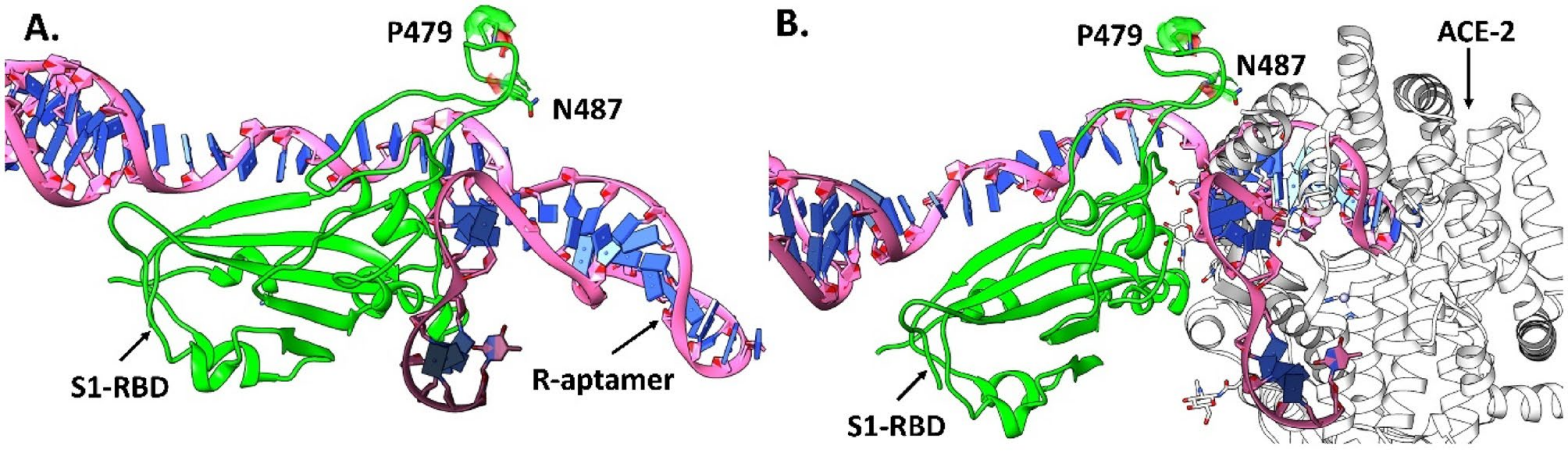
Lowest energy binding pose of (**A**) aptamer R (pink) docked at the RBD (green) of the S1-protein near residues P479 and N487 and (**B**) the superimposition of the docked RBD-aptamer R complex with the ACE2 receptor (grey) [PDB ID: 6MOJ] would interfere with normal S1-RBD binding to ACE2.

While the experimentally determined aptamer R efficiently inhibited SARS-CoV-2 through RBD binding, a better understanding of the specific binding site and important intermolecular interactions present in the aptamer-RBD system can be useful for future rational designs. Molecular docking studies of aptamer R with the S1 protein of SARs-CoV-2 performed here predicted binding to occur near the receptor binding motif (RBM) residues P479 and N487 of the S1 protein (Fig. 8A). Superimposing the most favorable docked pose of the RBD-aptamer R complex with the co-crystallized complex structure of the S1-ACE2 receptor (PDB ID: 6M0J) suggested that binding of the R aptamer at the RBM should interfere with the binding of the SARS-CoV-2 S1-RBD with the human ACE2 receptor (Fig. 8B). Interestingly, out of the top 10 docked poses of aptamer R to S1-RBD, it was observed that three poses were nearly identical (as they bind very close to the N487 and P479 residues) and adopted a conformation which may interfere with the binding of ACE2 receptor with S1-RBD in the presence of R aptamer (Fig. S6A). The remaining seven binding poses of R aptamer did bind at the RBD but not near N487 or P479. Nevertheless, superimposition with the S1-RBD-ACE2 complex suggests that all docking conformations should interfere with the binding of S1-RBD to the ACE2 receptor (Fig. S6B). Previous studies have shown that N487 of S1-RBD forms strong electrostatic interactions with Y83, Q324, Q325, E329, and N330 of the ACE2 receptor^28,45^. Hence, designing an aptamer that binds near the N487 or P479 residues may weaken or inhibit the binding of S1-RBD with the ACE2 receptor.

**Figure 9:**
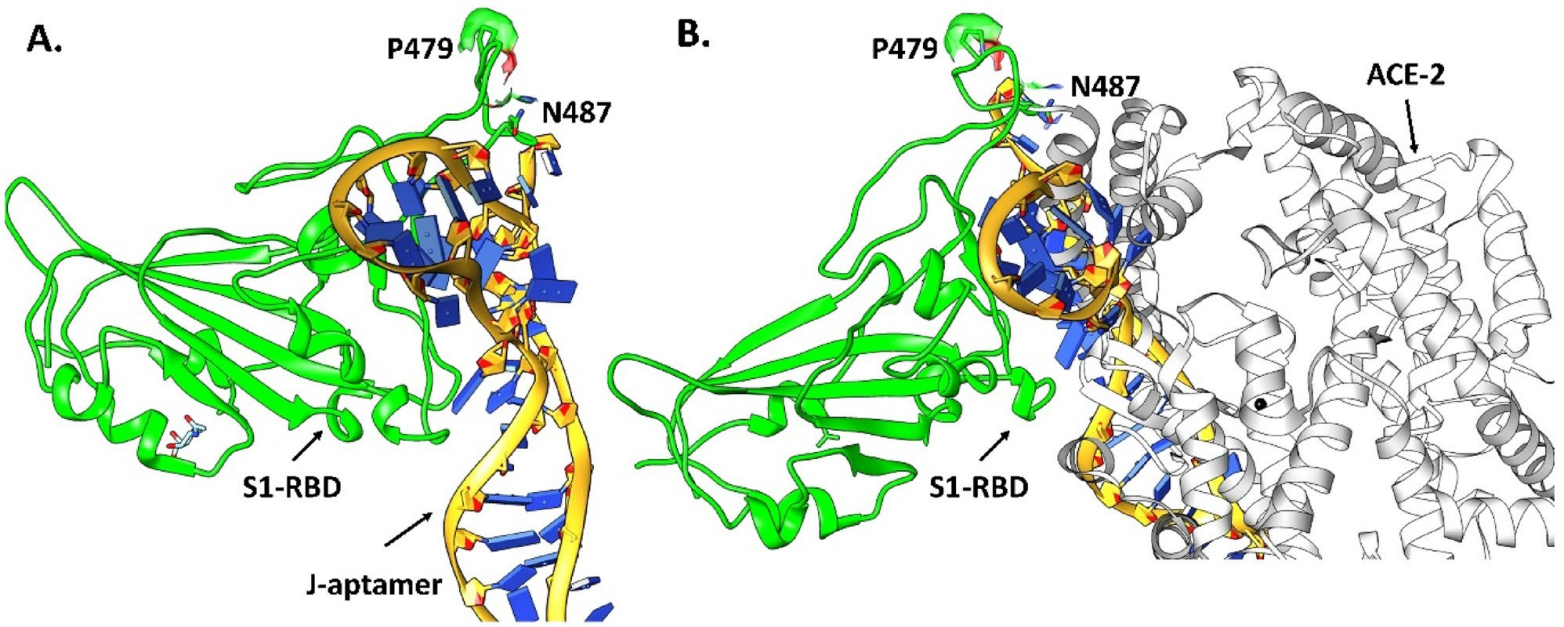
The most favorable docked pose of J aptamer (yellow) at the RBD (green) of S1-protein, where (**A**) shows that J aptamer binds at the RBM near the residue P479 and N487 of S1 protein and (**B**) shows that the selected binding pose of J aptamer would interfere with binding of the ACE2 receptor as indicated by superimposition of the docked complex (RBD-aptamer R) with the ACE2 receptor (grey) [PDB ID: 6MOJ].

As aptamer J has the same sequence as aptamer R, but without the forward and reverse primer regions, molecular docking was performed for aptamer J to assess the impact of the added primer region. Aptamer J was also predicted to bind close to residues P479 and N487 in the RBM of S1-RBD. However, the binding conformation of aptamer J was very different from aptamer R despite their similarities in sequence as R and J were bound vertically and perpendicular to the RBD, respectively (Figs. 8A and 9A). The most probable reason behind this difference may be the smaller length of aptamer J as compared to R. Nevertheless, superimposing the S1-RBD-aptamer J complex with the S1-RBD-ACE2 co-crystallized structure (PDB ID 6M0J) indicates that this compound should impede binding between S1-RBD and the ACE2 receptor (Fig. 9B). This observation was strongly supported by 5 out of the top 10 binding poses which also bind at the RBM and overlap with coordinates of the ACE2 receptor (Fig. S7A). While the remaining 5 docked poses were observed to bind at the RBD, they were far away from the residues P479 and N487 and only one pose (shown in pink in Fig. S7B) appears to interfere with the binding of the ACE2 receptor.

### Molecular dynamics simulations of the binding mechanism of aptamer R and J

**Figure 10:**
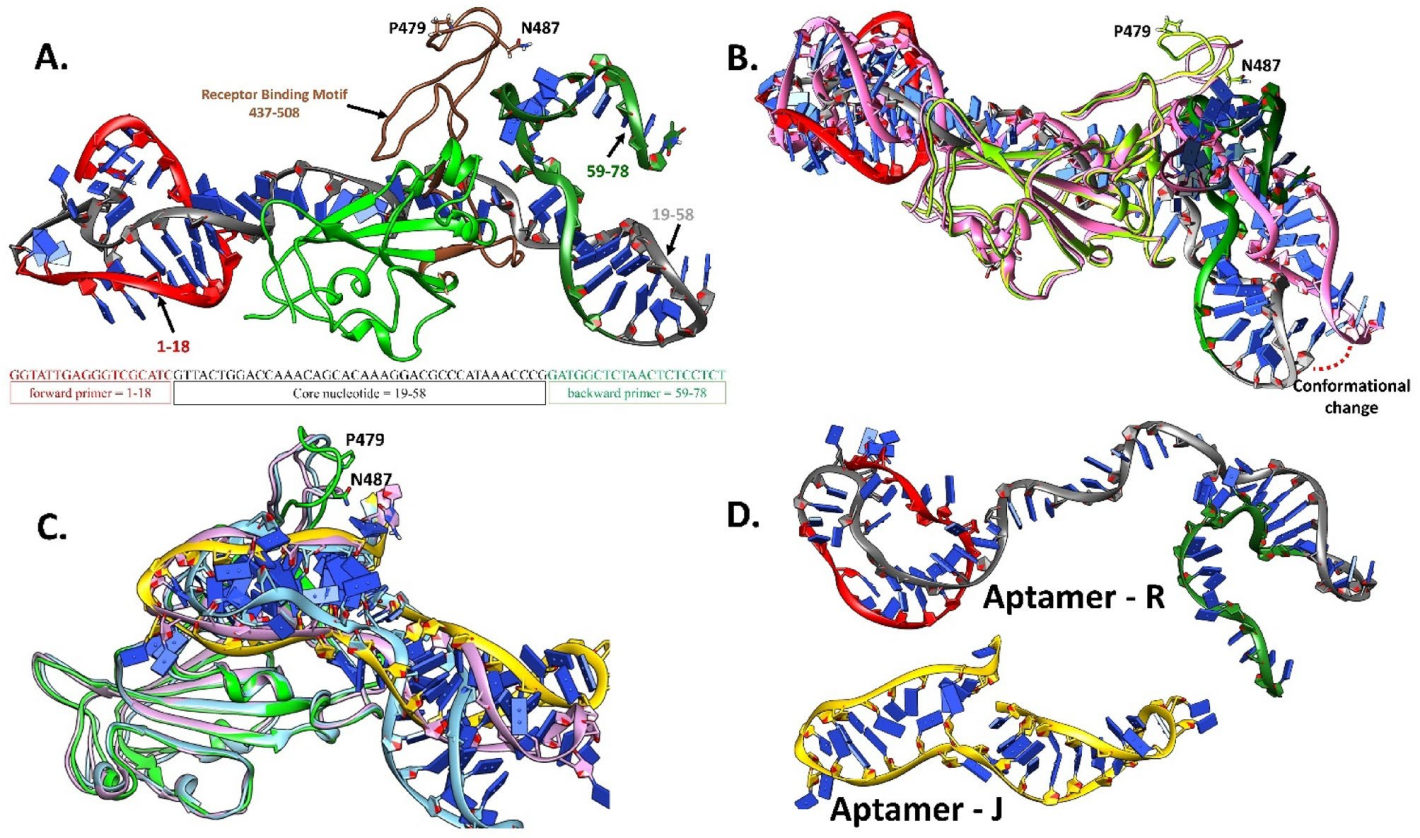
(**A**) The most dominate cluster structure (83.5%) from 300 ns of MD simulation of the aptamer R-S1-RBD complex, the R aptamer is composed of a forward primer region (1–18) [red], central core nucleotide (19–58) [grey], and reverse primer region (59–78) [olive-green] bound to S1-RBD (light-green) near the receptor binding motif (brown) that encompasses residue P479 and N487. (**B**) Highlights the conformational change occurring at the central core region of aptamer R when comparing the most dominate cluster with the initial docked complex (pink). (**C**) Comparison between the most dominate cluster of S1-RBD (light-green) bound with aptamer J (yellow) (55.2%), the second most dominate cluster (22.5%) (pink), and the initial docked pose (blue). (**D**) Conformational changes for aptamers R and J.

Biding affinity assays and molecular docking studies, while informative, lack the capability to provide detailed insight into prevalent interactions or potential conformational changes in the RBD protein that arise from aptamer binding. Hence, molecular dynamic (MD) studies of aptamers R and J in complex with S1-RBD were carried out. Root-mean-square deviations (RMSDs) of the backbone protein atoms within S1-RBD regions were examined (Figs. S1–S3) to understand the time scale required to stabilize the protein structure in response to aptamer binding. RMSD analysis showed that the aptamer R-S1-RBD complex reached equilibrium after 50 ns and aptamer J-S1-RBD took ~ 150 ns despite the shorter sequence in J compared to R (Fig. S2). Cluster analysis for each system showed that the aptamer R-S1-RBD complex had only one major conformation present with an 83.5% population over 300 ns of MD simulation, whereas the aptamer J-S1-RBD complex existed primarily in two major conformations with populations of 55.2% and 22.5%. MD simulations of aptamer R-S1-RBD identified a conformational change that occurred in the RBM (residues 437–508) with the central core region of the aptamer R moving parallel to the RBM (Fig. 10A,B). A similar reorganization of the RBM was observed for the most dominate cluster (55.2%) of aptamer J-S1-RBD from the MD simulations, but the second largest cluster (22.5%) retained a RBM conformation similar to the initial docked complex (Fig. 10C). In addition, comparison of the central core regions of the S1-RDB complexes found the central core region adopted a folded structure when bound with aptamer J in contrast to aptamer R where only ~ 50% of the sequences were folded leading to a less rigid structure (Fig. 10D). The lack of primer regions in aptamer J reduces the sequence to 40 (from 78 in R) and consequently has one of the lowest numbers of unpaired base pairs compared to R and other aptamers (Table S3). Interestingly, while aptamer R has the highest number of paired base pairs, it has retained a highly flexible central core region (Fig. 10D) compared to the more rigid structure in aptamer J.

### Calculated binding affinities for aptamers R and J

To follow up on these flexibility differences between the aptamers, binding energies (Δ*G*_bind_) were computed using MD simulations in conjunction with the Poisson–Boltzmann surface area continuum solvation (MM/PBSA) method for aptamers R and J bound to the S1-RBD of SARS-CoV-2. The Δ*G*_bind_ values found that aptamer R binds ~ 3 times stronger to the RBM of S1-RBD in comparison to aptamer J (Table 3). To make sense of this substantial preference for aptamer R binding to S1-RBD, the individual energy contributions that compose the Δ*G*_bind_ energies were examined. The calculations found the van der Waals energy (*E*_vdW_) and non-polar contributions *G*_np_ to the solvation energy changes to be nearly identical for both aptamers. This suggests that the high selectivity observed between the aptamers is derived from the electrostatic energy change (*E*_el_) and free energy polar contribution (*G*_pol_) terms, which were both ~ 2 times greater for aptamer R compared to aptamer J (Table 3).

**Table 3:** Decomposition of the free energy of binding (ΔG_bind_ in kcal/mol) for the binding of aptamers R and J bound at the S1-RBD of SARS-CoV-2.

Complex	*E*_vdW_	*E*_el_	*G*_pol_	*G*_np_	ΔG_bind_
R-RBD	− 155.3 ± 31.3	− 3078.3 ± 266.3	3134.2 ± 272.2	− 17.5 ± 2.8	− 116.9 ± 29.3
J-RBD	− 138.2 ± 19.1	− 1579.2 ± 179.0	1689.2 ± 182.5	− 14.9 ± 1.8	− 43.15 ± 22.4

E_vdW_ = van der Waals energy, E_el_ = electrostatic energy, G_pol_ and G_np_ = polar and nonpolar contributions to the solvation-free energies, respectively.

Analysis of the computed electrostatic interactions between the aptamers and S1-RBD found a greater hydrogen bonding population for the aptamer R-S1-RBD complex compared to aptamer J (Table S4). To clarify, aptamer R formed a large hydrogen bond population of ~ 75% with the glucose moiety attached to S1-RBD in comparison to aptamer J which formed a reduced population of ~ 27%. In addition, out of 25 different hydrogen bonds observed between aptamer R and S1-RDB, 17 of them were formed from the aptamer R central core region (nucleotide base pairs: 19–58). The binding of aptamer R induced a conformation change in both the RBM and the central core region of aptamer R which may suggest an induced fit mechanism.

**Figure 11:**
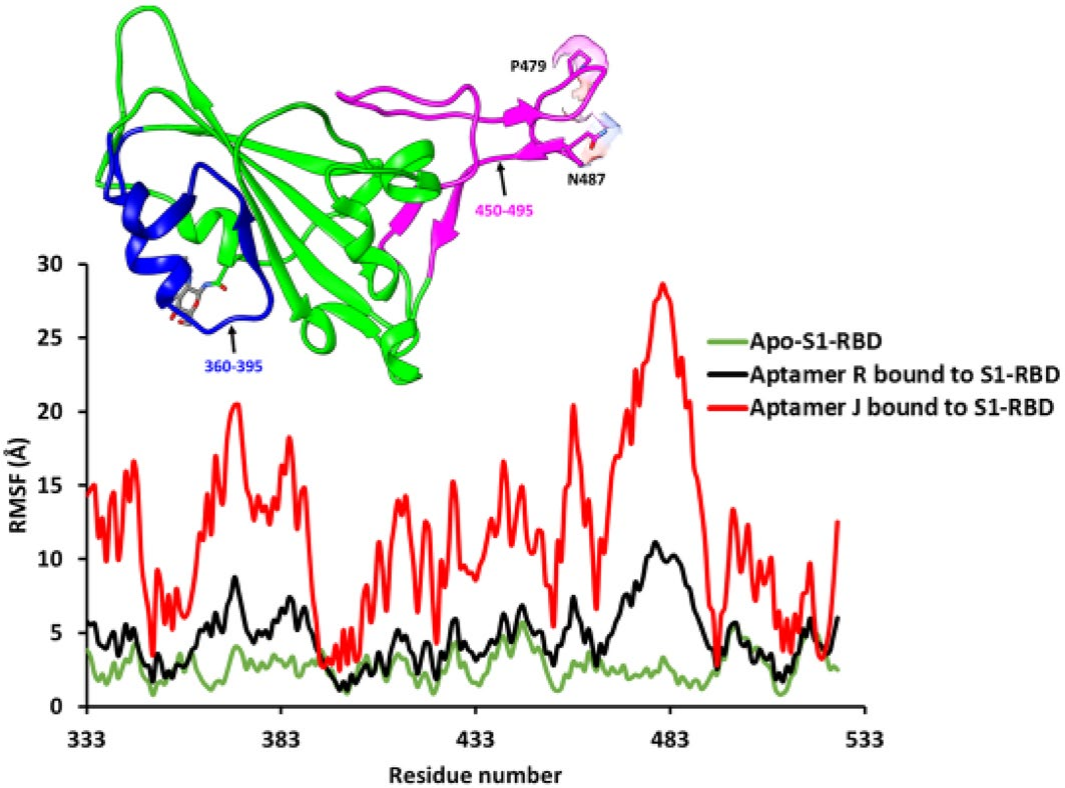
Root-mean-square fluctuations (RMSFs) by residue relative to the average structure over 300 ns simulation trajectory for the S1-RBD protein backbone atoms (N, C_α_ and C) in the apo state [green] and holo state (bound with aptamer R [black] or J [red]).

While the binding free energy results offer one explanation for the preferred selectivity of aptamer R, additional atomic insight can be derived by numerically estimating the changes in residue conformation arising from aptamer binding. Accordingly, the root-mean-square-fluctuation (RMSF) of the complexes were used to examine which regions of protein diverged the most from the average structure. The RMSF analysis found that the aptamer J-S1-RBD complex had very high fluctuations compared to R, suggesting a high mobility for the system (Fig. 11). Conversely, the RMSF fluctuations computed for the aptamer R-S1-RBD system resembled the unbound state (apo) of S1-RBD with the exception of the high fluctuations observed around the aptamer binding region composed of residues 450–495. In addition, the % change in RMSF showed that same region in S1-RBD had the highest magnitude of localization effect in response to the binding of aptamer R (Fig. S3A) and that the aptamer R primer region became more flexible (Fig. S3B). This suggests that the presence of the forward and reverse primer regions may be essential in making aptamer R more selective towards S1-RBD.

## Conclusion

Accurate, reliable, and economically viable theranostic kits fulfilling the purpose of rapid diagnosis and therapeutic need remain incredibly important for controlling the spread of COVID-19 infection. In response, we have developed aptamers against SARS-CoV-2 virus using SELEX which hold potential for application in the diagnostics and treatment of SARS-CoV-2 infection. The appropriate regions (target) in the virus must be recognised by a suitable moiety to develop a diagnostic system. Considering the phylogenetic history of coronaviruses, a viral oligopeptide target was selected based on a multiple sequence alignment of spike proteins from MERS-CoV, SARS-CoV-1, and SARS-CoV-2. Further, specificity of the detection system was enabled by selecting only two amino residues as the conserved region (CR) while two flanked variable regions (eight amino acid each) were selected on either side of CR. During SELEX screening, aptamer R was identified as a potent binder of RBD and showed excellent sensitivity for SARS-CoV-2 at a very low concentration of 0.3 μM. Encouraged by in vitro assays that showed exceptional binding with RBD, the antiviral potential of the screened aptamers was analyzed. Aptamer R showed the best activity (95.4% inhibition) whereas aptamer J (same central sequence as aptamer R but lacking flanked primer regions) showed the second-best inhibition (82.5%) against the SARS-CoV-2 virus. Moreover, using a pseudovirus based entry assay it was confirmed that the aptamer R can specifically inhibit the entry of the SARS-CoV-2 spike pseudotyped viruses in cells expressing ACE2 receptors (HEK293T-ACE2). Despite these encouraging results, the exact binding site between the aptamers and RBD was unknown. Consequently, in silico studies were performed to provide a better understanding. Molecular docking calculations found that both aptamers R and J were preferentially bound near residues P479 and N487 in the RBM of the S1-RBD protein. Superimposition of the most favorable docked poses with an experimental crystal structure of S1-RBD suggested that both aptamers should interfere with binding to the ACE2 receptor. More rigorous molecular dynamics simulations were used to compute free energies of binding and found that aptamer R binds ~ 3 times stronger to S1-RBD than aptamer J. The simulations reported enhanced electrostatic interactions present upon binding aptamer R that were derived from a large hydrogen bond population of ~ 75% with the glucose moiety attached to S1-RBD in comparison to aptamer J which formed a reduced population of ~ 27%. The calculations noted that binding of aptamer R in S1-RBD prompted a conformation change in both the RBM and the central core region of aptamer R that suggests an induced fit mechanism may be at play. In summary, our newly developed aptamer has great potential for the diagnosis of SARS-CoV-2 at a very low concentrations and possesses the ability to act as a potent entry inhibitor (greater than 90%).

### Supplementary Information

Supplementary Information.

## Data Availability

The datasets generated for this study can be accessed at NCBI Bio Project: PRJNA975735 and Sequence Read Archive (SRA): SRR24718814, SRR24718812, SRR24718815, SRR24718813 and SRR24718816.
