# Supplementary material for: SELEX based aptamers with diagnostic and entry inhibitor therapeutic potential for SARS-CoV-2
Source: Sci Rep. 2023 Sep 4;13:14560. doi: 10.1038/s41598-023-41885-w (PMC10477244; doi:10.1038/s41598-023-41885-w)
Supplement: Supplementary file 1 — Supplementary Information. [file 41598_2023_41885_MOESM1_ESM.docx]

**SELEX based aptamers with diagnostic and entry inhibitor therapeutic potential for SARS-CoV-2**

Sayanti Halder^1#^, Abhishek Thakur^2#^, Supriya Suman Keshry^3,5^, Pradip Jana^1^, Divyanshi Karothia^3^, Indrani Das Jana^4^, Orlando Acevedo^2^, Rajeeb K. Swain^3^, Arindam Mondal^4^, Soma Chattopadhyay^3^, Venkatesan Jayaprakash^1^, Abhimanyu Dev^1*^

^1^Department of Pharmaceutical Sciences and Technology, Birla Institute of Technology, Mesra, Ranchi, 835215, Jharkhand, India.

^2^Department of Chemistry, University of Miami, Coral Gables, FL, 33146, United States.

^3^Institute of Life Sciences, Bhubaneswar, 751023, Odisha, India.

^~~4~~^School of Bioscience, Indian Institute of Technology Kharagpur, Kharagpur, 721302, West Bengal, India.

^5^School of Biotechnology, Kalinga Institute of Industrial Technology (KIIT) University, Bhubaneswar, Odisha, India.

^#^Authors SH and AT have contributed equally.

*Corresponding author: Abhimanyu Dev Corresponding address: Department of Pharmaceutical Sciences & Technology, Birla Institute of Technology, Mesra, Ranchi-835215, India. Tel.: +91-651-2275210; fax: + 91-651-2275401. Email: [abhimanyudev@bitmesra.ac.in](mailto:abhimanyudev@bitmesra.ac.in)


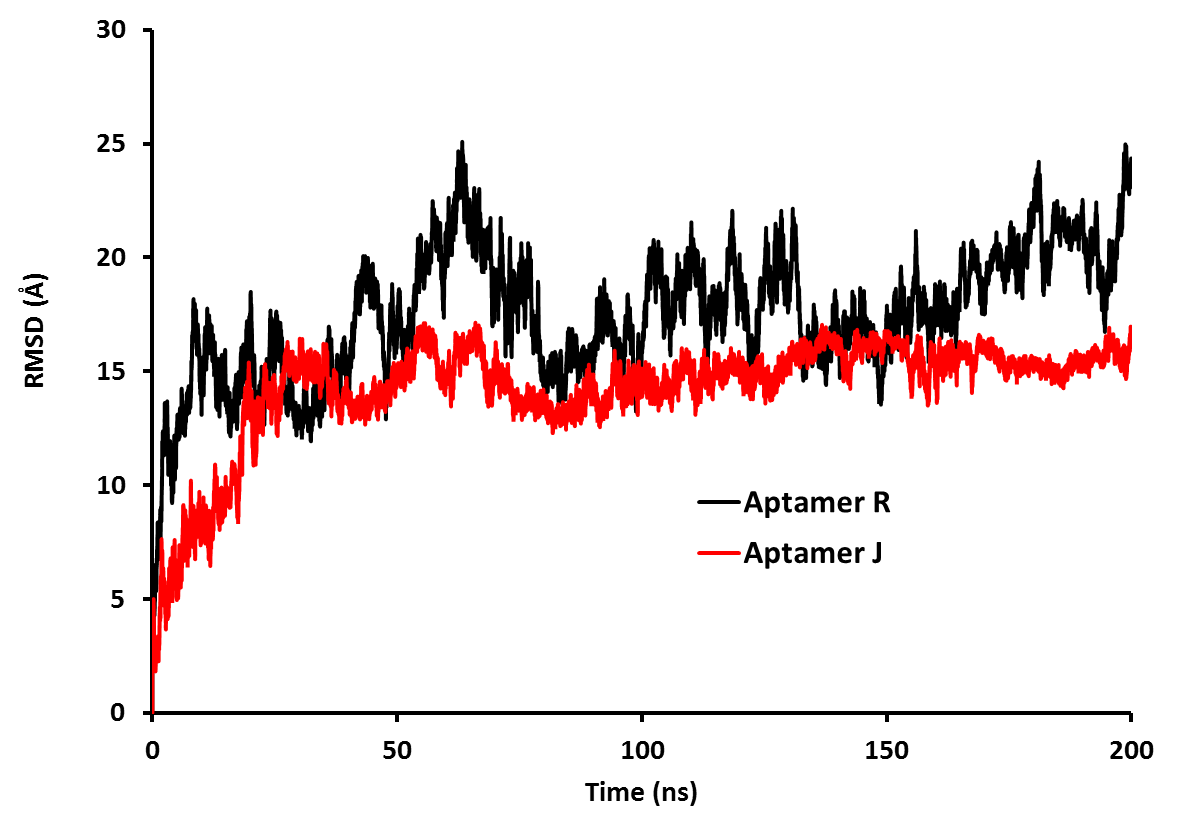


**Figure S1:** Root-mean-square deviation (RMSD) of the aptamers C atoms for the respective simulations relative to the first frame.


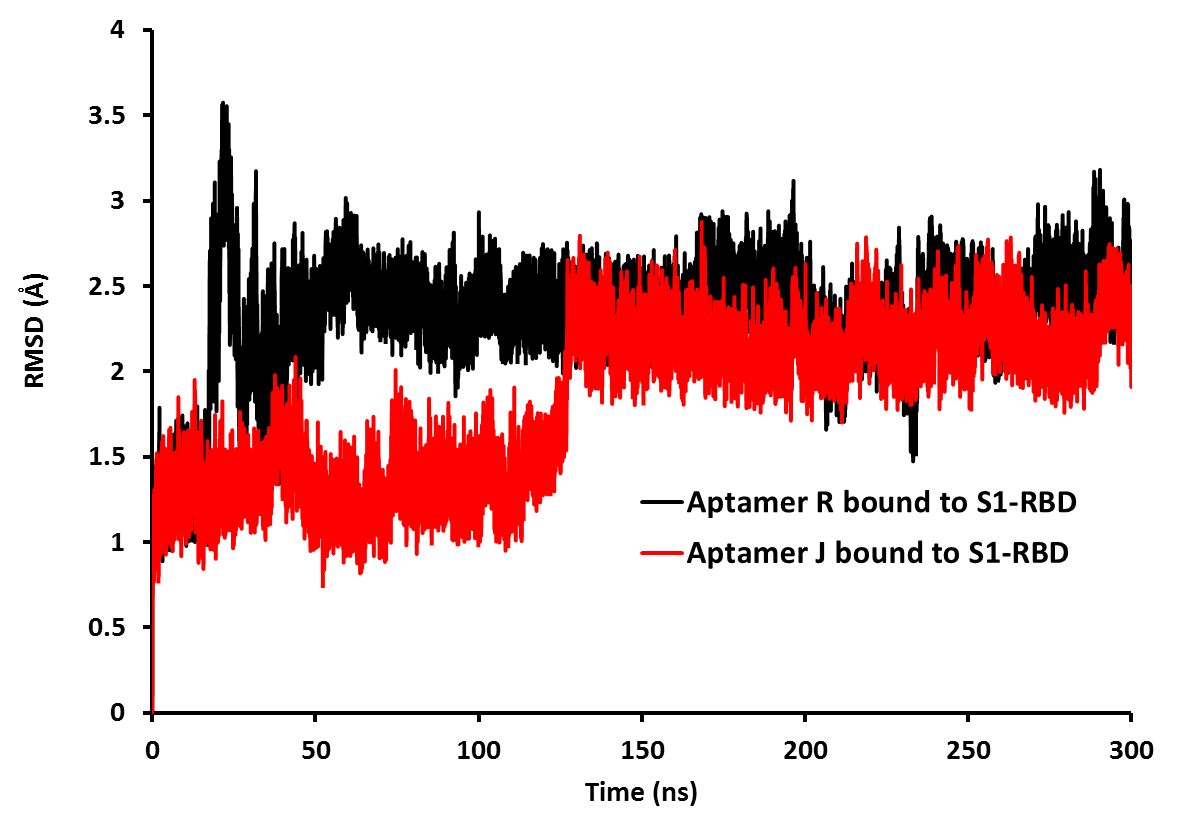


**Figure S2:** Root-mean-square deviation (RMSD) of the S1-RBD protein backbone atoms (N, Cα and C) for the respective simulations relative to the first frame.


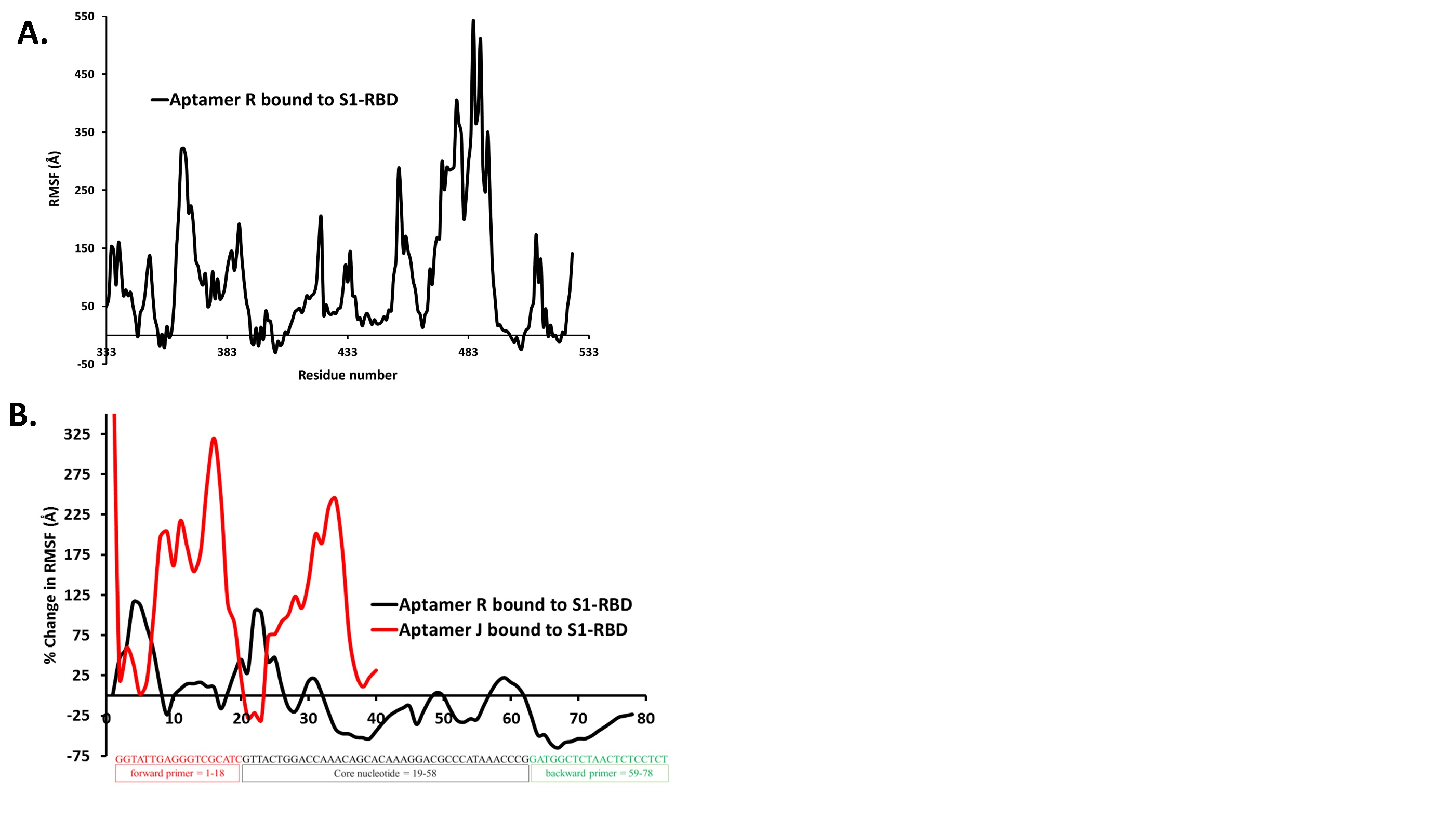


**Figure S3:** Showing the average percent change in root-mean-square fluctuations (RMSF) of A) the S1-RBD backbone atoms (N, Cα and C) upon binding of aptamer R and B) aptamer R and J in response to their binding to S1-RBD of SARS-CoV-2. Where a positive change in RMSF shows that residues have become more localized and a negative change is indicative of enhanced fluctuations upon aptamer binding.


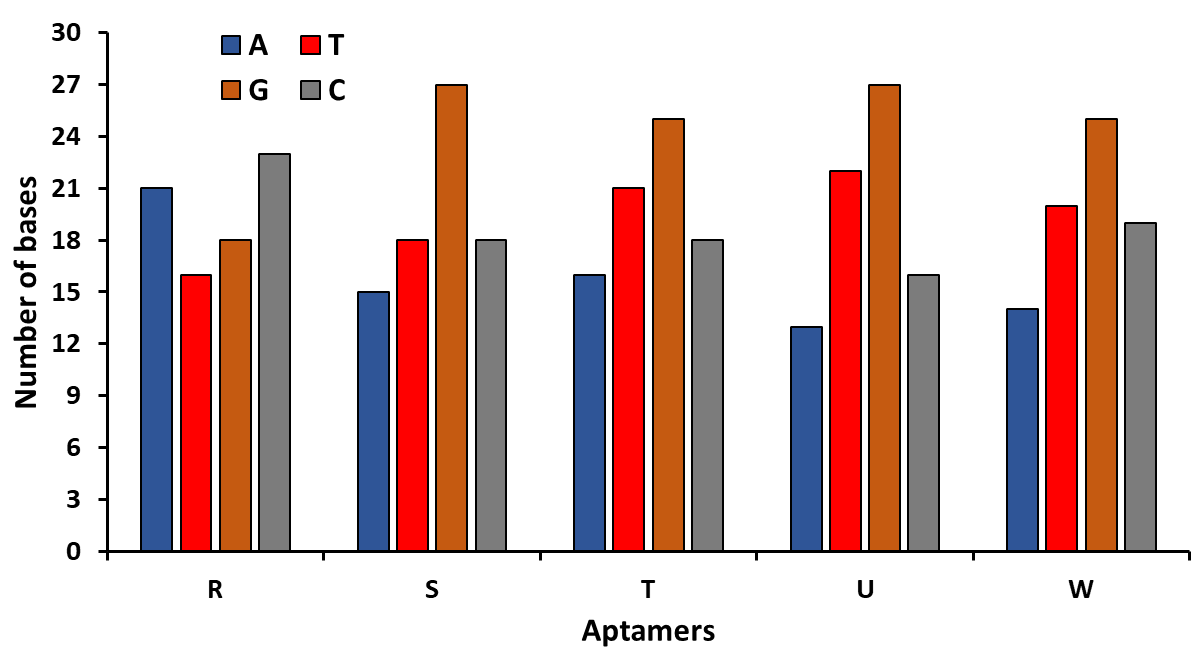


**Figure S4:** Illustrates total numbers of bases in selected five aptamer sequences.


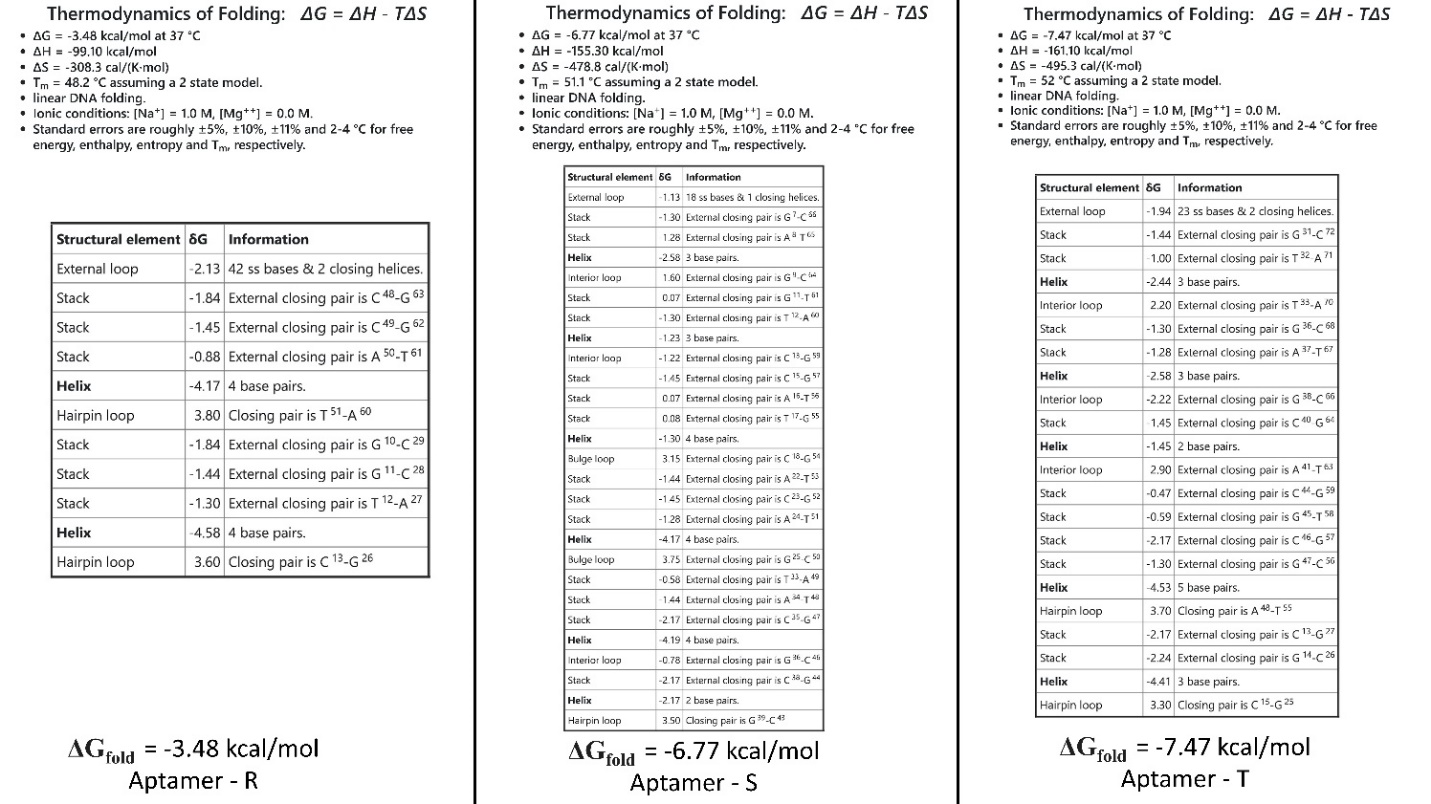


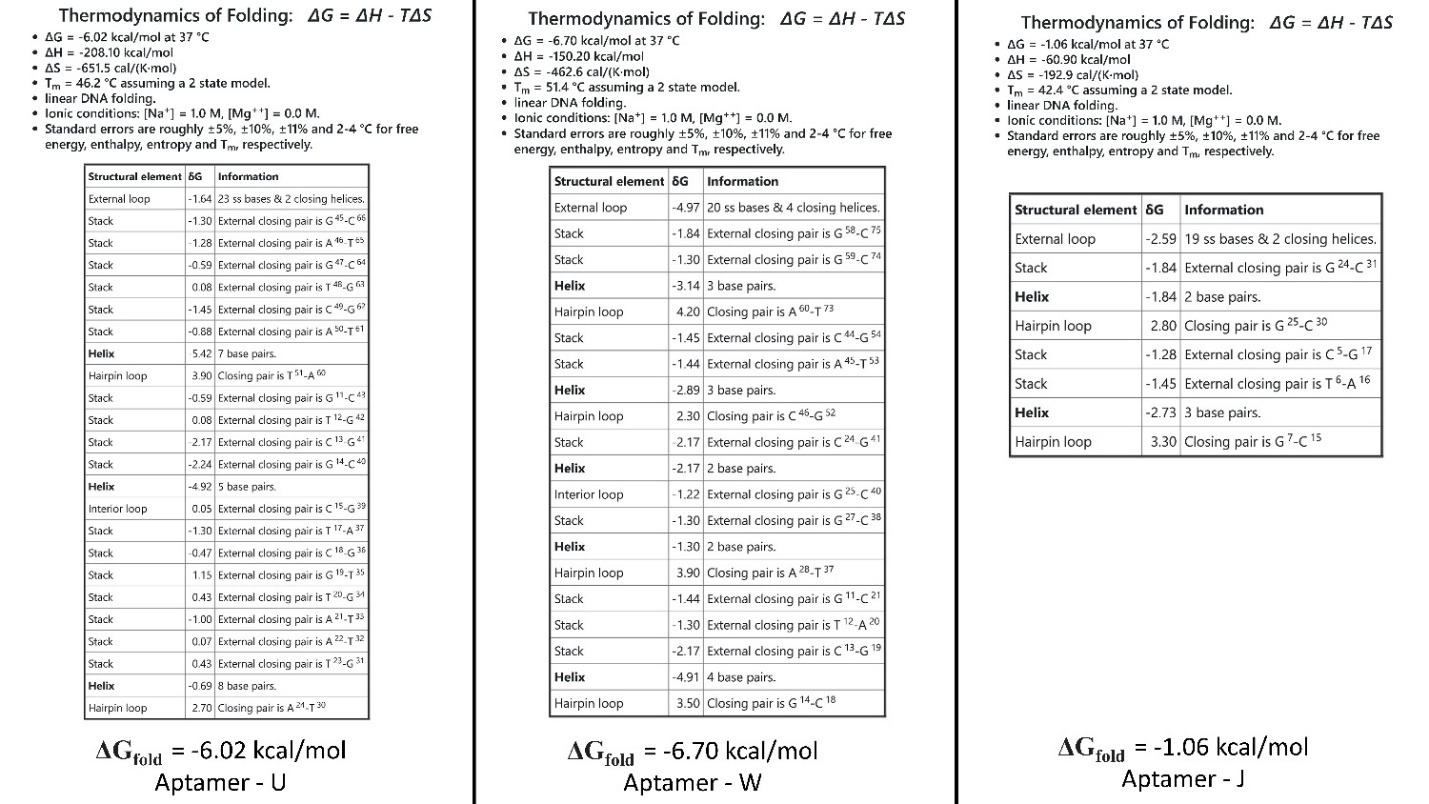


**Figure S5:** Illustrating the thermodynamics of folding for aptamers R, S, T, U, W, and J. predicted by mfold server^1^.


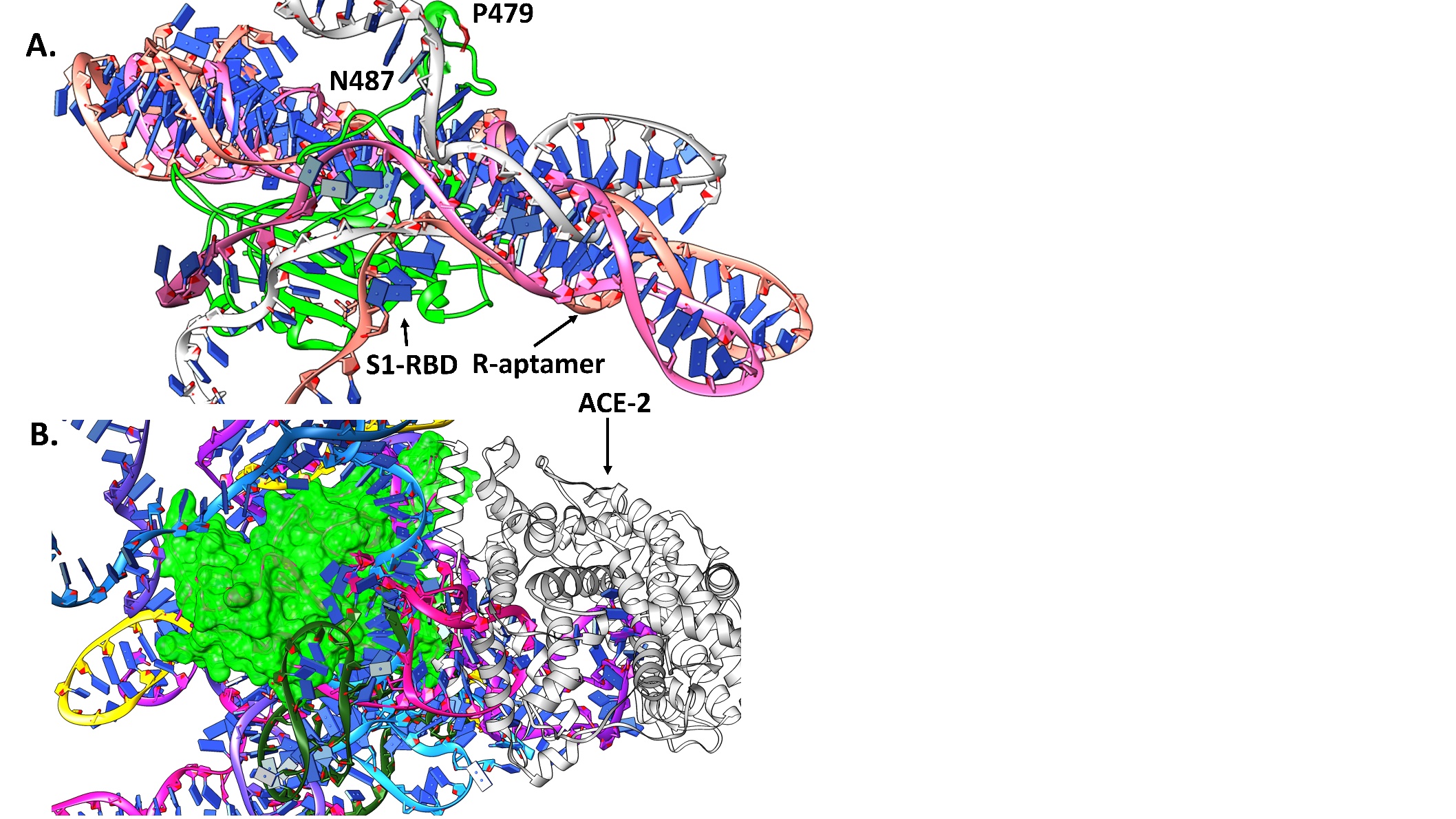


**Figure S6:** Illustrating the top 10 binding poses of R aptamer docked at the RBD (green) of S1-protein, where (A) shows that out of the top 10 poses, 3 R aptamer binds in a very identical fashion (where they bind at the RBM and will not allow the binding of S1-RBD with human ACE-2 receptor) and (B) shows that remaining 7 binding poses binds little far away from residue P479 and N487 but it binds in a way that will interfere with binding of ACE-2 receptor as observed by superimposing the docked complex (RBD - R aptamer) with ACE-2 receptor (grey) [PDB ID: 6MOJ].


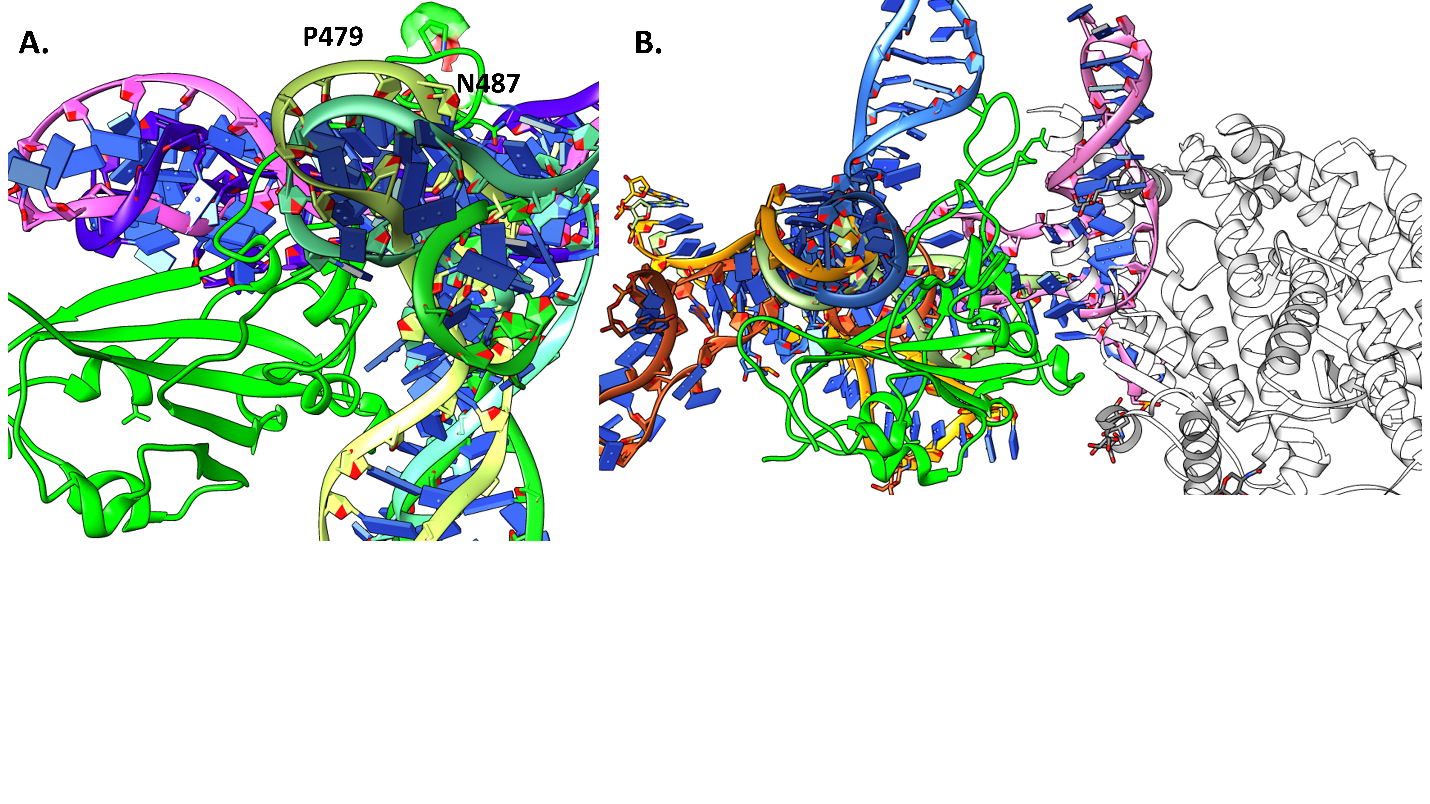


**Figure S7:** Illustrating the top 10 binding poses of J aptamer docked at the RBD (green) of S1-protein, where (A) shows that out of the top 10 poses 5 J aptamer binds in a very identical fashion (where they bind at the RBM and will not allow the binding of S1-RBD with human ACE-2 receptor) and (B) shows that remaining 5 binding poses does not bind at the RBM and expect 1 pose (pink) no other will interfere with binding of ACE-2 receptor as observed by superimposing the docked complex (RBD - R aptamer) with ACE-2 receptor (grey) [PDB ID : 6MOJ].


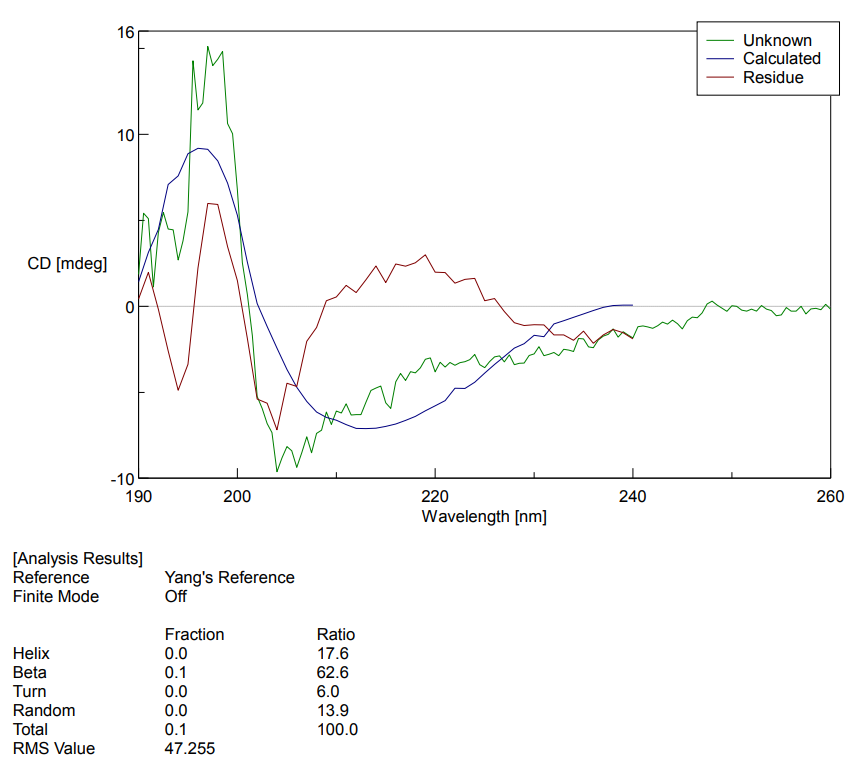


**Figure S8:** Figure showing circular dichroism (CD) spectra of viral oligopeptide.

**Table S1:** Illustrates the folded 2D structure in Vienna format, folding energy (ΔG) in kcal/mol, and number of bases for a few potent aptamers.

|  | Dot-bracket representation | ΔG_fold_ (kcal/mol) | # BP |
| --- | --- | --- | --- |
| R | .........((((............))))..................((((........))))............... | -3.48 | A's = 21, C's = 23, G's=18, T's = 16 |
|  | .........((((............))))............((....)).....((.....))............... | -3.23 |  |
|  | .........((((............))))............((....))........(((............)))... | -2.6 |  |
| S | ......(((.(((.((((...((((.......((((.((...)).)))))))))))).)))..)))............ | -6.77 | A's = 15, C's = 18, G's=27, T's = 18 |
|  | ......(((.(((.((((...((((..(((...)))((((....)))).)))))))).)))..)))............ | -6.56 |  |
|  | ............(((...........))).......((((....)))).........(((............)))... | -6.16 |  |
|  | ..........(((((.(((...............))).)))))..............(((............)))... | -5.99 |  |
|  | ......(((((((.((((...((((.......((((.((...)).)))))))))))).)))............)))). | -5.96 |  |
| T | ............(((.........)))...(((..(((.((..(((((......)))))...)).))).)))........ | -7.47 | A's = 16, C's = 18, G's=25, T's = 21 |
|  | ......((((..(((.........)))...(((..(((.((..(((((......)))))...)).))).)))...)))). | -7.34 |  |
|  | ........................((((.(((......))).))))....((.((....)).))................ | -7.22 |  |
|  | ........................((((.(((......))).))))............(((....)))............ | -6.47 |  |
|  | ........................((((.(((......))).))))............((((..........)))).... | -6.53 |  |
| U | ..........(((((.((((((((.....)))))))).))))).(((((((........)))))))............ | -6.02 | A's = 13, C's = 16, G's=27, T's = 22 |
|  | ............(((.........)))........((((.....(((((((........))))))).......)))). | -5.39 |  |
|  | ......((((..(((.((((((((.....)))))))).)))...(((((((........))))))).......)))). | -5.04 |  |
| W | ..........((((...))))..((.((........)).))..(((.....)))...(((............)))... | -6.7 | A's = 14, C's = 19, G's=25, T's = 20 |
|  | ............(((..((.(((.....)))...))..)))..(((.....)))...(((............)))... | -6.25 |  |
|  | ..........((((...))))....((((.....((((.(..((((.....)))).).)))).........))))... | -6.09 |  |
|  | ..........((((...)))).....((((((..((((.(..((((.....)))).).))))..))..))))...... | -6.04 |  |
| J | ....(((.......)))......((....))........ | -1.06 | A's = 15, C's = 13, G's=8, T's = 4 |
|  | ((..(((.......))).))...((....))......... | -0.23 |  |
|  | .........((............))............... | -0.22 |  |

**Table S2:** Based on the sequence number of A-T and G-C pairs that can be formed was calculated.

|  | R | S | T | U | W |
| --- | --- | --- | --- | --- | --- |
| A-T | 16 | 15 | 16 | 13 | 14 |
| G-C | 18 | 18 | 18 | 16 | 19 |
| Total | 34 | 33 | 34 | 29 | 33 |

**Table S3:** Based on the sequence number of unpaired bases in the sequence was calculated

|  | R | S | T | U | W | J |
| --- | --- | --- | --- | --- | --- | --- |
| A | 5 | 3 | 0 | 0 | 0 | 11 |
| T | 0 | 0 | 5 | 9 | 6 | 0 |
| G | 0 | 9 | 7 | 11 | 6 | 0 |
| C | 5 | 0 | 0 | 0 | 0 | 5 |
| Total | 10 | 12 | 12 | 20 | 12 | 16 |

**Table S4:** Hydrogen bond % occupancy formed between aptamers (R and J) and S1-RBD of SARS-CoV-2.

| **Aptamer** | **Hydrogen Bond Acceptor** | **Hydrogen Bond Donor** | **%** | **Distance (Å)** | **Angle** |
| --- | --- | --- | --- | --- | --- |
| **R** | A39@OP2 | ASN450@NDH | 88.12 | 2.8 | 165.2 |
|  | C38@OP(1+2) | SER349@OG | 97.92 | 2.6 | 163.3 |
|  | C38@OP1 | SER349@NH | 63.94 | 2.8 | 153.8 |
|  | A39@OP2 | ASN448@N2H | 72.63 | 2.8 | 164.7 |
|  | ASN450@OD1 | A40@N6H | 49.78 | 2.8 | 154.7 |
|  | C66@OP2 | ARG403@N2H | 39.95 | 2.8 | 154.8 |
|  | C66@OP(1+2) | ARG403@N1H | 66.93 | 2.8 | 154.5 |
|  | A27@OP2 | NAG@O(3+4)H | 75.45 | 2.6 | 165.0 |
|  | A37@OP1 | ASN354@N2H | 36.86 | 2.8 | 163.2 |
|  | A37@OP1 | ARG466@N1H | 46.36 | 2.8 | 157.9 |
|  | G46@OP2 | GLN498@NEH | 26.98 | 2.8 | 160.4 |
|  | C47@OP1 | THR500@OGH | 26.16 | 2.7 | 163.0 |
|  | C38@O4 | ARG346@N2H | 25.58 | 2.9 | 146.8 |
|  | C66@OP2 | TYR453@OH | 25.45 | 2.7 | 160.9 |
|  | C66@OP1 | TYR505@OH | 22 | 2.7 | 162.6 |
|  | GLY447@O | G42@N2H | 18.28 | 2.8 | 157.3 |
|  | C70@OP1 | SER477@OGH | 18.23 | 2.8 | 157.3 |
|  | A31@OP2, O5 | ARG357@N1H | 41.49 | 2.8 | 156.2 |
|  | G46@OP2 | GLY446@N1H | 15.62 | 2.9 | 163.7 |
|  | T71@OP1 | SER477@OGH | 26.17 | 2.7 | 165.2 |
|  | VAL445@O | G43@N(1+2)H | 24.43 | 2.9 | 152.0 |
|  | G10@OP1 | ARG357@N2H | 12.9 | 2.8 | 160.5 |
|  | C45@O2 | ASN501@N2H | 12.08 | 2.9 | 158.7 |
|  | ASN448@OD1 | A41@N6H | 12.00 | 2.9 | 151.2 |
|  | G11@OP1 | ASN334@NDH | 21.98 | 2.8 | 161.4 |
| **J** | A35@OP2 | TYR489@OH | 73.32 | 2.7 | 165.0 |
|  | G1@O5 | SER501@NH | 65.34 | 2.9 | 158.7 |
|  | A36@N(1+7) | GLN493@NEH | 97.33 | 2.9 | 160.7 |
|  | A35@OP2 | SER447@OGH | 49.03 | 2.6 | 165.3 |
|  | A22@OP2 | ARG403@NH | 83.5 | 2.8 | 160.9 |
|  | LEU492@O | C37@N4H | 38.19 | 2.9 | 160.7 |
|  | C37@O2 | TYR449@OH | 30.53 | 2.8 | 162.9 |
|  | A34@OP2 | GLY476@NH | 28.58 | 2.8 | 157.5 |
|  | G25@OP2 | GLN409@NEH | 28.58 | 2.8 | 156.4 |
|  | G24@N7 | LYS417@NH | 36.2 | 2.9 | 162.4 |
|  | G25OP(1+2) | ARG408@N1H | 85.74 | 2.8 | 156.5 |
|  | A35@OP2 | ASN487@N2H | 14.74 | 2.8 | 159.5 |
|  | G17@OP2 | NAG@O(3+4)H | 27.29 | 2.6 | 165.7 |
|  | A4@N1 | GLN498@NEH | 12.43 | 2.9 | 164.1 |
|  | PHE490@O | A36@N6H | 10.66 | 2.9 | 148.1 |
|  | LEU455@O | A34@N6H | 10.24 | 2.9 | 151.1 |

**Estimation of Dissociation constant (Kd):**

Aptamer R (10 µL) was conjugated with fluorescent carbon dot (100 µL) in the weight ratio of 1:1. Here carbon dot was used as a fluorescence probe. The carbon dot used here was synthesized previously in our lab. Similar type of carbon dot was synthesized in our lab and used in colorectal cancer targeted drug delivery (10.1016/j.mtcomm.2022.104590). The carbon dot used here has the excitation maxima (Ex_max_) of 320 nm. The aptamer R and carbon dot were added together and incubated for 2 h at 4°C, 50 rpm in an incubator shaker. RBD conjugated NiNTA (RBD@NiNTA) was prepared by incubating His-tagged RBD (1 µL of 1 µg/ µL) with NiNTA slurry (10 µL) at 4°C for 3 h. Unbound RBD was washed by applying magnet. Further, various concentrations (0, 30, 60, 120, 240, and 480 nM) of aptamer-carbon dots conjugated solution was added to RBD@NiNTA in PBS and incubated at 37°C for 2h. Activation of carbon dots-aptamer R solution was done by heating at 90°C for 10 min then cooling at 0°C for 20 min. Subsequently, the carbon dots-aptamer R- RBD@NiNTA system was washed and diluted up to 50 µL with PBS. Fluorescence emission was checked at Ex_max_ of 320 nm using Spectramax i3X plate reader. The emission maxima for the system were obtained at 370 nm. Further, the fluorescence (relative fluorescence unit: RFU) for the different concentrations of aptamer R conjugated with carbon dots was noted (Table S5) and plotted to determine the Kd value. The obtained Kd value for the aptamer R was 125.26 nM.

**Table S5:** Table showing respective fluorescence intensity at different aptamer R concentrations.

| **Aptamer R Concentration (nM)** | **Fluorescence intensity (RFU)** |
| --- | --- |
| 0 | 0 |
| 30 | 22146 |
| 60 | 326283 |
| 120 | 402186 |
| 240 | 1048282 |
| 480 | 804430 |

**References:**

1. Zuker, M., Mfold web server for nucleic acid folding and hybridization prediction. *Nucleic Acids Res* 2003, *31* (13), 3406-15.
